# Supplementary material for: Robust synchronization of coupled circadian and cell cycle oscillators in single mammalian cells
Source: Mol Syst Biol. 2014 Jul 15;10(7):739. doi: 10.15252/msb.20145218 (PMC4299496; doi:10.15252/msb.20145218)
Supplement: Supplementary file 1 — Supplementary Information [file msb0010-0739-sd1.pdf]

Supplemental Information

Robust synchronization of coupled circadian and cell cycle oscillators in single mammalian cells

Jonathan Bieler, Rosamaria Cannavo, Kyle Gustafson, Cedric Gobet, David Gatfield, and Felix Naef

The Institute of Bioengineering, School of Life Sciences, Ecole Polytechnique Federale de Lausanne (EPFL), AAB 040 Station 15, Lausanne, CH-1015, Switzerland

Contents

I. Cell segmentation and tracking 3

A. Cell segmentation 3

B. Cell tracking 8

II. Stochastic phase model 9

A. Likelihood function 9

B. Boundary conditions 13

C. Numerical approximation 13

D. Parameter optimization 15

E. Validation and parameters identifiability 19

III. Phase inference using a Hidden Markov Model 22

A. Validation 25

IV. Synchronization indices 27

V. Granger causality 29

A. Nuclear area and cell cycle phase 29

B. Granger Causality 34

References 35

VI. Supplemental figures (S1 to S13) 36

I. CELL SEGMENTATION AND TRACKING

Imaging circadian signal can be challenging as cells will go through high and low fluorescence intensity during the circadian cycle. As a result an image can be overexposed for some cells (saturating the detector) and underexposed (cells being hard to distinguish from background) for other cells. In addition to this time dependent change in the signal, there is also significant cell-to-cell variability in the fluorescence intensity.
In order to mitigate this problem we took three images with different exposure times at each time point. This permits collection of unsaturated images for signal quantification at low exposure time, while being able to segment low intensity cells by combining the different images as explained in the next section.

A. Cell segmentation

The segmentation is an important step in the data analysis, as segmentation errors will lead to short traces and reduce the overall quality of the data. Most segmentation errors occurs either around mitosis due to the very low YFP signal during nuclear envelope breakdown, and during cytokinesis as cells can have dramatic changes in shape and texture. Since we are interested in division times it is important for us to minimize segmentation errors linked to divisions. Another source of error is the phase of the circadian cycle when the YFP signal is low, during which some cells can become hard to detect automatically. In order to minimize these segmentation errors and assure the quality of our dataset we did a manual validation of all automatically segmented frames.
The segmentation is composed of three main steps:

1. Preprocess and combine images
2. Automatic segmentation
3. Manual validation

In the first step the background from the three images obtained using different exposure times (30ms, 50ms, 80ms) is corrected by subtracting the low frequencies from the image. This allows removal of the large scale illumination inhomogeneities and obtains an even

background. Then the images are denoised using a median filter. This allows averaging of neighboring pixels while keeping the edges relatively sharp. Finally the three images are normalized and averaged (Figure M1B).

In the second step the combined images are segmented. The segmentation procedure is straightforward: the image is convolved with a family of cell-like filters (Figure M2B). The resulting images are then averaged (Figure M2C) and converted to binary masks using an adaptive threshold (Figure M2D, left). The binary images are cleaned using morphological operations (Figure M2D, right). Finally an automatic correction is done by splitting adjacent cells that have been recognized as one object. An example of an automatically segmented image is shown in Figure M1C.

In the third step the segmented images are manually corrected and validated using a custom Matlab tool shown in Figure M3. An example of a typical correction is shown in Figure M1D.

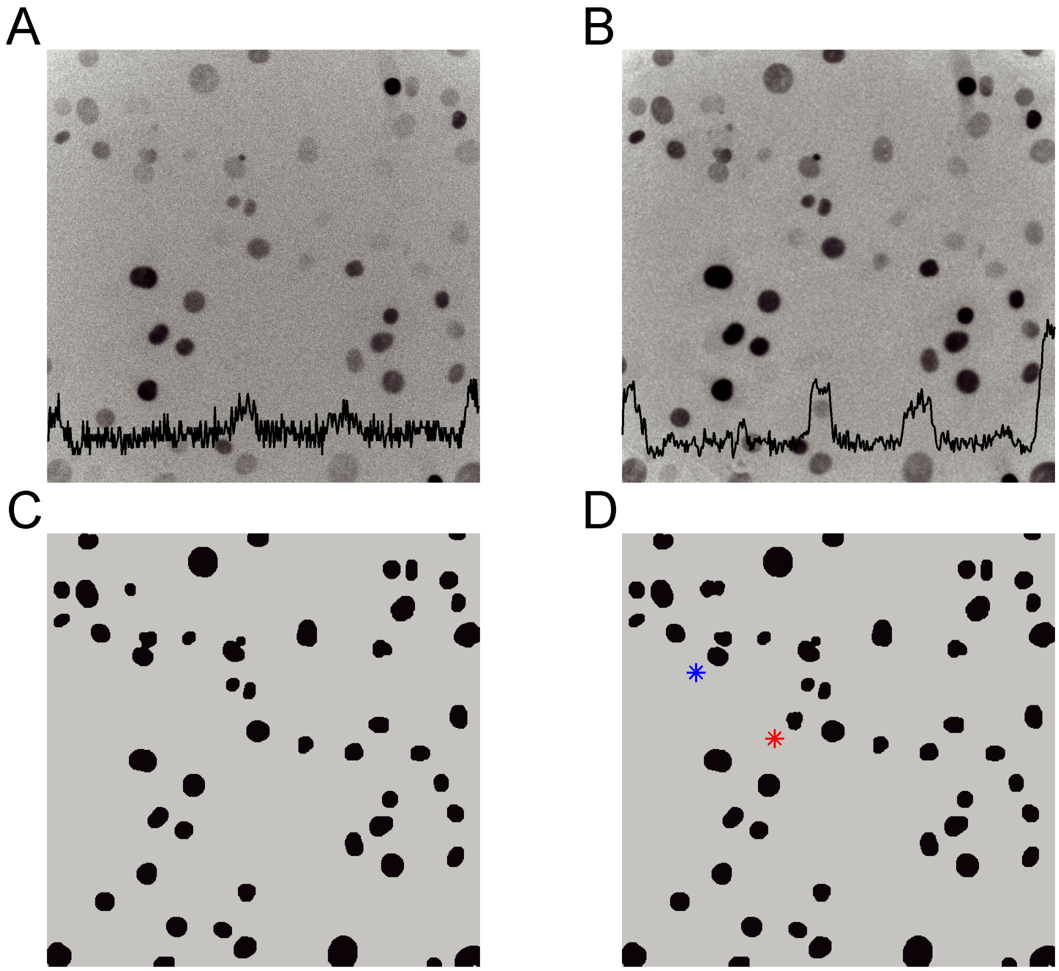

Figure M1: **Cell segmentation overview.** **A.** Raw image used for quantification. For ease of visualization the image is shown with false color map that enhances contrast. **B.** Preprocessed image ready for segmentation. The black line plot in A and B is the normalized image intensity taken on a single row. **C.** Segmented image after the automatic segmentation. **D.** Segmented image after the manual validation. Two errors were corrected, in blue two adjacent cells that were recognized as one object in panel C were separated, in red a missing cell was added.

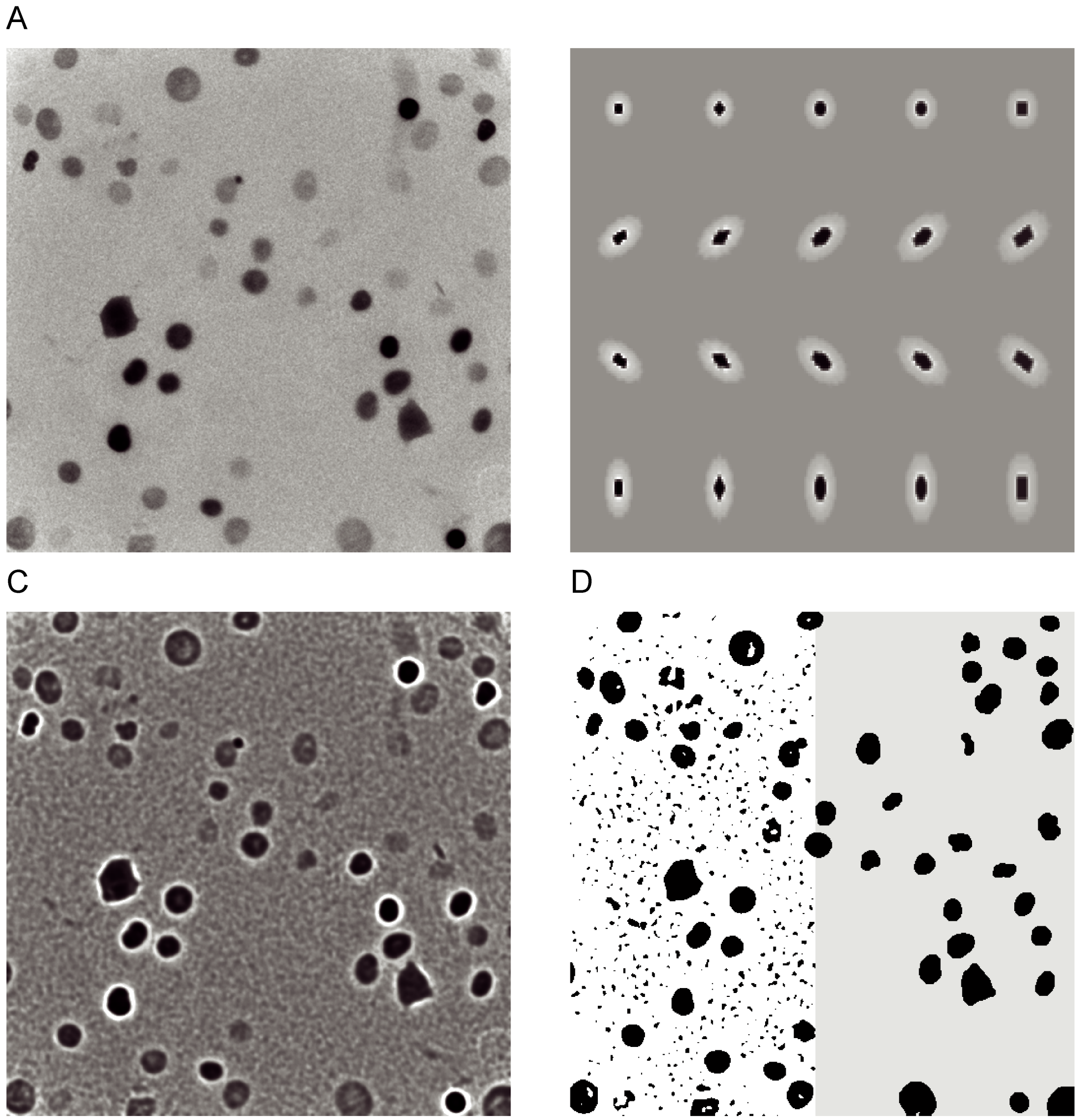

Figure M2: **Segmentation steps A.** Preprocessed image before segmentation. **B.** Small collection of cell-like filters. **C.** Filtered image. **C.** Thresholded image, the left part shows the image before cleaning with morphological operations while the right part shows the cleaned image.

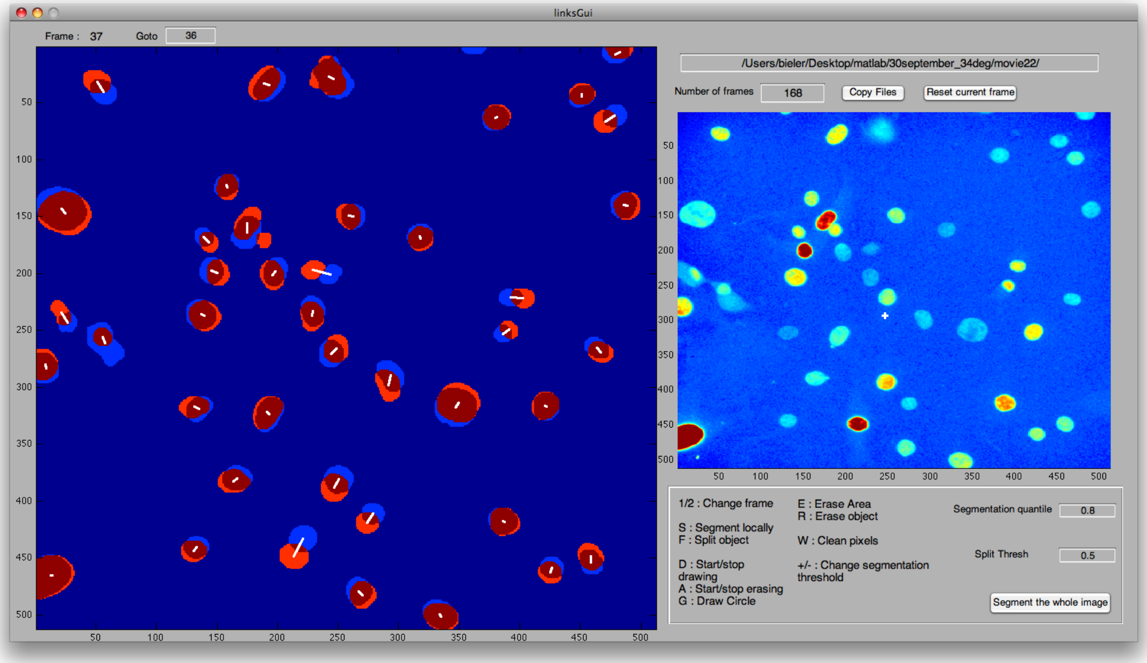

Figure M3: **Segmentation validation tool.** The left panel shows both the current segmented frame and the previous one. Red areas correspond to part of the image that are recognized as objects in the two frames, orange to ones that are only present in the current frame, and light blue to the ones that are only present in the previous frame. The white lines represent which objects are linked by local tracking between the two frames. The left panel shows the image before the segmentation. The user is able to execute a series of operations on the current segmented frames to correct automatic segmentation errors.

B. Cell tracking

Once the images are segmented, detected cells need to be tracked in time in order to obtain time traces. We used a standard tracking algorithm [6] to follow our cells. This algorithm links objects frame by frame based on a certain list of features (here we used distance and fluorescence intensity) before performing a global assignment. Importantly it supports divisions. As our cells enter and leave the camera view and new cells appear with division, we obtained a collection of time traces of different lengths. In our 37 °C dataset we obtained traces of 55 hours on average and about 25% of traces span the whole recording time of 72 hours.

Even if the tracking algorithm can reliably detect new objects generated by divisions, there is a significant error in the mother-to-daughter assignment. For that reason, and in order to simplify our analysis we decided not to use lineage information in our main analysis. More precisely, when a new cell is generated by mitosis, a new independent trace is created at the division time to keep track of the daughter cell.

II. STOCHASTIC PHASE MODEL

The simplest and most general way to model two coupled noisy phase oscillators is to reduce the system to two coupled phase variables. The only underlying assumption is that amplitude fluctuations in these oscillators decouple from the phase dynamics. We can then describe the system as a two dimensional stochastic differential equation (SDE). The phase of the circadian cycle is given by θ. In the absence of coupling (coupling function $F_1 = 0$) the circadian phase is a Brownian motion with drift, where the drift term is given by 2π over the circadian period T_1 and the noise term by $\sigma_1 \mathrm{d}W_t$ where W_t is a Wiener process. The phase of the cell cycle ϕ follows a similar and independent Brownian motion.

$$\mathrm{d}\theta_t = \frac{2\pi}{T_1}\mathrm{d}t + F_1(\theta_t, \phi_t)\mathrm{d}t + \sigma_1 \mathrm{d}W_t \tag{1}$$

$$\mathrm{d}\phi_t = \frac{2\pi}{T_2}\mathrm{d}t + F_2(\theta_t, \phi_t)\mathrm{d}t + \sigma_2 \mathrm{d}Y_t \tag{2}$$

The periodic function $F_1(\theta, \phi)$ represents the influence of the cell cycle on the circadian clock: positive regions of $F_1(\theta, \phi)$ speed up the circadian clock while negative ones slow it down.

Here we used to following parametrization for the coupling function: $F_1 = K_1 G_1(\theta, \phi) + K_2 G_2(\theta, \phi)$ where G_i's are 2D Gaussians with diagonal covariance matrices. This means that the cell cycle can possibly interact with the circadian clock at different phases and have different effects (speed up and slow down) depending on the sign of K_i. The coupling function F_2 is written in the same way: $F_2 = K_3 G_3(\theta, \phi) + K_4 G_4(\theta, \phi)$. An illustration of such functions is shown in Figure S6.

This parametrization allows for flexible coupling functions that can account for different scenarios, while remaining relatively economical in terms of number of parameters.

A. Likelihood function

Our dataset is a collection of sequences of time-ordered events (circadian peaks and divisions) corresponding to individual cell traces.

We denote the successive circadian peaks of a particular trace as $p_1, .. p_N$ where each $p_i \in (0, \Delta t, .. t_{max})$ represents the timing of the circadian peak with respect to the beginning of the trace. Similarly we denote the successive divisions as $d_1, .. d_M$.
For simplicity of notation, when the type of event is unspecified we will denote event i as e_i so that the time ordered sequence of N events corresponding to a particular cell can be written as $S_N = (e_1, .. e_N)$ and its sub-sequence $(e_1, .. e_{N-1})$ as S_{N-1}.
In the context of our phase model we interpret our data as the first-hitting time of the corresponding oscillator:

$$p_i = \inf_{t \geq 0}(t : \theta(t) = 2\pi \mid \theta(p_{i-1}) = 0)$$
$$d_i = \inf_{t \geq 0}(t : \phi(t) = 2\pi \mid \phi(d_{i-1}) = 0)$$

Then the probability of a sequence S_N can be recursively decomposed as:

$$\begin{aligned} P(S_N) &= P(e_1, .. e_N) \\ &= P(e_N | e_{N-1}, .. e_1) P(e_N - 1, .. e_1) \\ &= P(e_N | e_{N-1}, .. e_1) P(S_{N-1}) \end{aligned} \tag{3}$$

Since the phase of the relevant oscillator is by definition known exactly at event time, the probability of the event e_N depends on the last event of the same kind e_j plus all the events of the other kind that happened in-between:
$P(e_N | e_{N-1}, .. e_1) = P(e_N | e_{N-1}, .. e_j)$.
For example the probability of the sequence $S_4 = (d_1, p_1, d_2, p_2)$ is decomposed as:
$P(S_4) = P(p_2 | d_2, p_1, d_1) P(S_3) = P(p_2 | d_2, p_1) P(S_3)$.
As our measurements are invariant under time translation, we can rewrite the conditional probability $P(p_2 | d_2, p_1)$ as $P(p_2 - p_1 | d_2 - p_1, 0) = P(p_2 - p_1 | d_2 - p_1)$.
This quantity represents the probability distribution of a circadian interval $p_2 - p_1$ given that a division took place at time $d_2 - p_1$ after the first circadian peak. Figure M4 shows a scatter plot of theses two quantities measured in mouse fibroblasts. Figure M5 shows the conditional probability estimated from the model in a synchronized regime similar to the one

we observe in the data. For comparison this conditional probability is shown in Figure M6 for the same model with all coupling constants K_i's set to zero. Importantly the structures present in these distributions strongly depend on the coupling functions F_1 and F_2.

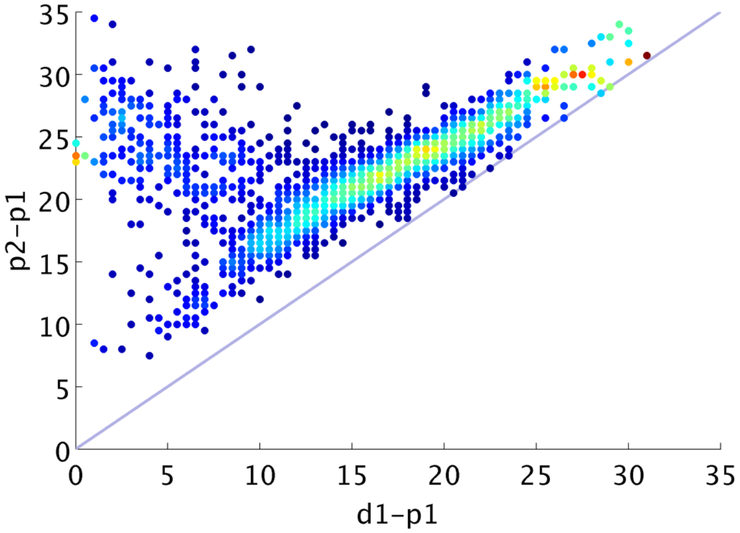

Figure M4: **Distribution of subintervals in** (p_1, d_1, p_2) **events.** The color intensity (blue: lowest, red: highest) represents the estimated conditional density of the data points (the integral on $p_2 - p_1$ is equal to one for each $d_1 - p_1$ values). Since we are plotting events of the type (p_1, d_1, p_2), the quantity $p_2 - p_1$ is by definition always larger than $d_1 - p_1$, so the data are confined above the diagonal. Note that the second peak takes place around 5 hours after the division (the data are shifted above the diagonal), and that this delay depends on the division time (the band is slightly curved). Note also that when the divisions take place early in the circadian cycle ($d_1 - p_1 < 10$ hours) then the 5 hour locking breaks down.

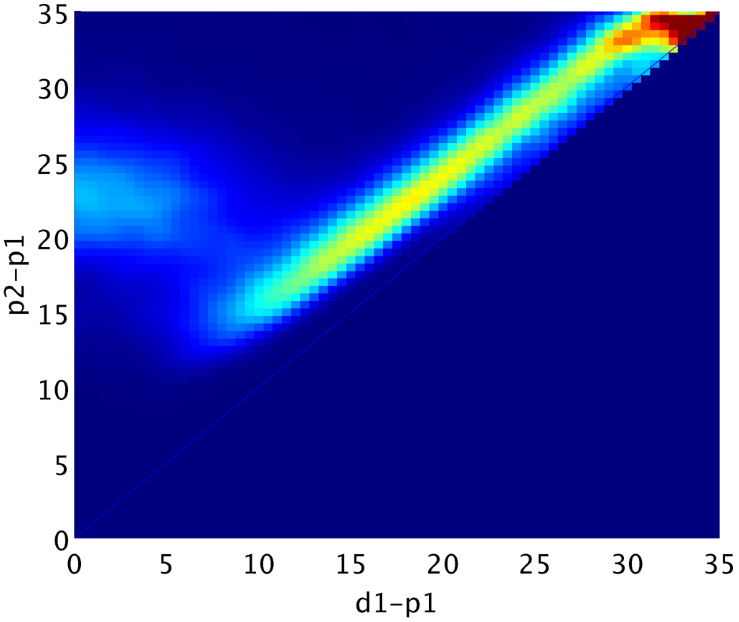

Figure M5: **Conditional probability** $P(p_2 - p_1, d_1 - p_1)$ **in a synchronized model.** Note that most of the features in Figure M4 are reproduced in the model.

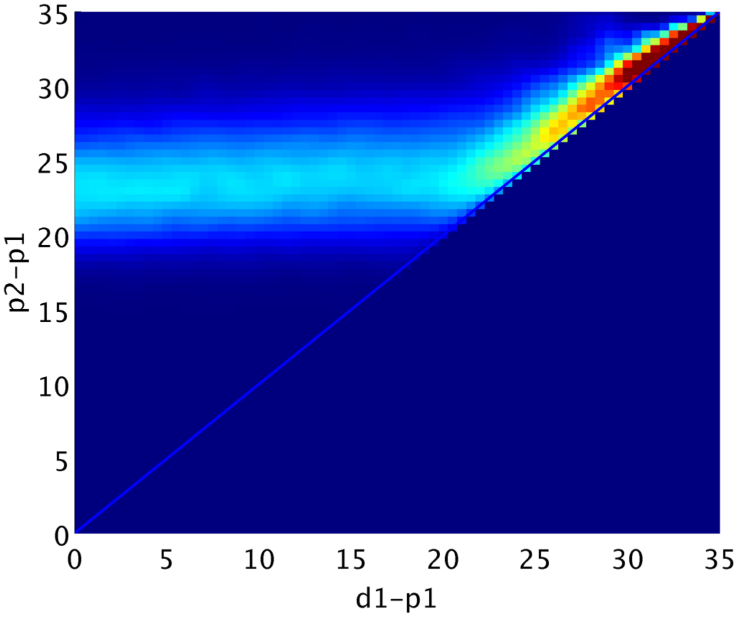

Figure M6: **Conditional probability in a model without coupling.** Note that in the absence of coupling between the two cycles, the circadian interval $p_2 - p_1$ is independent of the division time until the distribution comes close to the diagonal. Then the distribution get squeezed against the diagonal by the selection of time ordered events (p_1, d_1, p_2).

B. Boundary conditions

As we measure only finite time traces, we need to deal with boundary conditions when the recursive decomposition of sequence probability using Equation (3) reaches the end of a sequence.

For example, calculating the probability of the sequence $S_4 = (d_1, p_1, d_2, p_2)$ will eventually lead to the sub-sequence $S_2 = (d_1, p_1)$.

In this particular case we assume that an unobserved circadian peak p_0 took place somewhere before the beginning of the trace (time zero). We can then write the probability $P(S_2)$ as:

$$
\begin{aligned}
&P(d_1, p_1) = \int_{-\infty}^{0} P(p_0, d_1, p_1)P(p_0)\mathrm{d}p_0 \\
&= \int P(p_1|d_1, p_0)P(d_1, p_0)P(p_0)\mathrm{d}p_0 \\
&= \int P(p_1 - p_0|d_1 - p_0)P(d_1 - p_0)P(p_0)\mathrm{d}p_0 \\
&= \int P(p_1 - p_0, d_1 - p_0)P(p_0)\mathrm{d}p_0
\end{aligned}
$$

A natural distribution to use for $P(p_0)$ is a truncated inverse Gaussian (since it is related to the first passage time in a diffusion-drift process [3]) with mean $p_1 - T_1$ and variance $\sigma_1^2 T_1^3/(2\pi)^2$. Figure M7 shows the distribution $P(S_2)$ estimated from the model.

Other event types - e.g. (p_1, d_1) - are treated in a similar way.

C. Numerical approximation

The estimation of the conditional probability $P(p_2 - p_1|d_2 - p_1)$ is done by simulation. First we perform stochastic simulations of equation (1) with the initial condition $\theta(0) = 0$ (circadian peak at time zero) and $\phi(0) = \phi_0$, where ϕ_0 is a uniformly distributed random variable between 0 and 2π. The simulation is performed until $\theta(t)$ hits 2π. If $\phi(t)$ passed once through 2π in this interval we record these two hitting times as p_2 and d_2. The joint distribution $P(p_2,d_2)$ is then estimated on a reasonable support from a large number of such events using Gaussian kernel density estimation. The conditional probability $P(p_2|d_2)$ is finally computed from the joined distribution: $P(p_2|d_2) = P(p_2, d_2)/P(d_2)$ with $P(d_2) = \int P(p_2, d_2)\mathrm{d}p_2$.

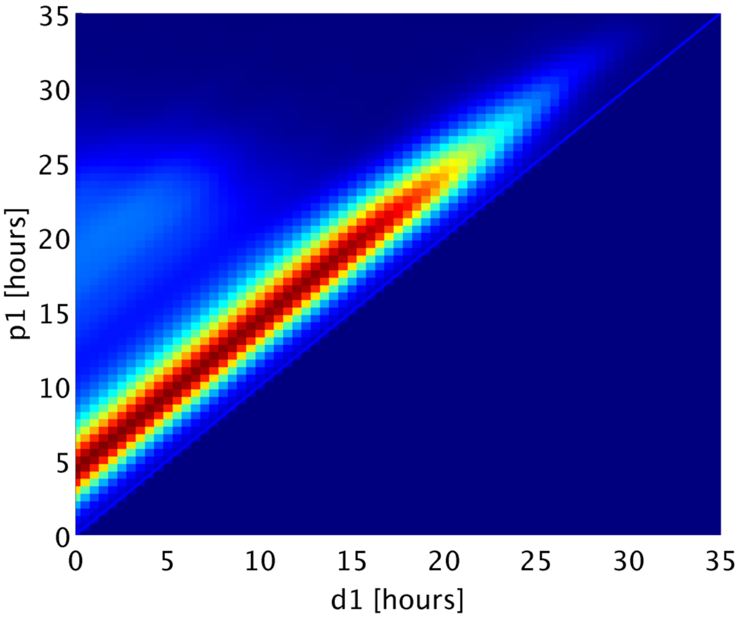

Figure M7: This distribution is similar to the one shown in Figure M5, but has been blurred in the diagonal direction by the integration over the unknown peak p_0. This reflects the lack of knowledge about the precise timing of this non-measured peak.

This conditional probability can then directly be used the evaluate the probability of our dataset. The same procedure is used for other types of events.

Note that our datasets consist mainly of events of the type (p_1, d_1, p_2), (d_1, p_1, d_2) and (p_1, p_2): these event types represent 94% of all our data points. More complicated sequences like (p_1, d_1, d_2, p_2) (two divisions during a circadian interval), represent only 3.5% of all data points. This allows us to neglect some of these rare events.

Finally our likelihood function for a parameter vector p is simply the multiplication of the probability of each sequence S_i:

$$L(\{S\}|p) = \prod_i P(S_i)$$

or more conveniently the sum of the log of the probabilities.

One drawback of using stochastic simulations to estimate our likelihood function is that this function itself is stochastic, but our optimization approach can suitably handle this.

D. Parameter optimization

When using the parametrization for the coupling function described above our model has a total of 24 parameters. The value of some of these parameters is fairly well known (e.g. the circadian period is close to 24 hours), while others are unknown. Thus we bounded our parameter space to constrain our parameters values to realistic intervals.

In order to find the parameters that maximize our likelihood function we used the Covariance Matrix Adaptation Evolution Strategy (CMA-ES) algorithm [4]. This algorithm is well suited for our optimization problem as it has global search properties and can deal with a large numbers of unknown parameters. Unlike some deterministic algorithms, it also performs reasonably well on stochastic likelihood functions.

For each condition we ran several independent optimizations using random initial conditions. A few optimization traces are shown in Figure M8. The parameters values found are shown in Table M1 and the coupling functions are shown in the main text.

As explained in the main text, the 37 °C and the Longdaysin data were fitted separately. The data at different temperatures were fitted together, assuming that the cell cycle period only is affected by the temperature.

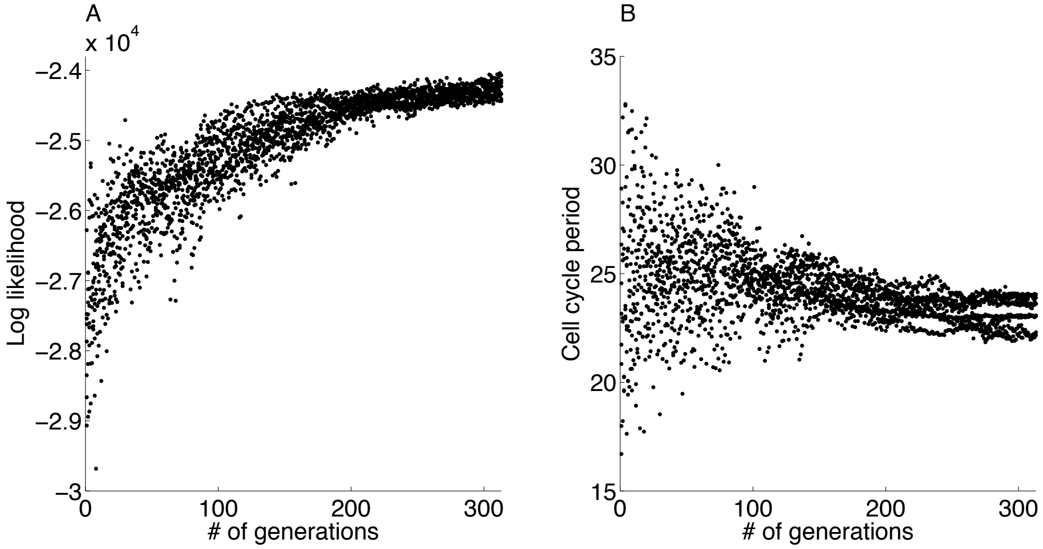

Figure M8: **Optimization traces. A.** The log likelihood is shown for a few different optimizations. **B.** The cell cycle period is shown as a function of the number of generations.

Condition	$T_1[h]$	$T_2[h]$	$\sigma_1[rad \times h^{-1/2}]$	$\sigma_2[rad \times h^{-1/2}]$
37 °C A	24.2 ± 0.5	23.75 ± 1.2	0.13 ± 0.03	0.37 ± 0.05
1 uM LD	27.5 ± 0.37	25.3 ± 1.32	0.18 ± 0.01	0.33 ± 0.02
3 uM LD	29.8 ± 0.79	30.7 ± 2.3	0.18 ± 0.008	0.31 ± 0.03
5 uM LD	32.2 ± 0.6	29.3 ± 2.5	0.18 ± 0.01	0.34 ± 0.03
37 °C	23.9 ± 0.6	20.5 ± 0.4	0.18 ± 0.02	0.33 ± 0.01
34 °C	idem	18.7 ± 1.0	idem	idem
40 °C	idem	24.4 ± 0.8	idem	idem

Table M1: **Estimated periods and noise values.** The values of the circadian period T_1, the cell cycle period T_2 and the respective noise coefficients are shown. The mean and standard deviations are computed on 10 to 30 independent solutions depending on the condition. 37 °C A refers to the 37 °C data that were fitted alone, while the three different temperatures at the end of the table were fitted together.

Condition	$K_1^+[rad \times h^{-1}]$	$\mu_\theta^+[rad]$	$\mu_\phi^+[rad]$	$\Sigma_\theta^+[rad]$	$\Sigma_\phi^+[rad]$
37 °C A	2.53 ± 0.95	3.90 ± 0.82	1.27 ± 1.43	0.70 ± 0.18	0.29 ± 0.13
1 uM LD	1.82 ± 0.57	3.65 ± 0.87	1.82 ± 2.12	0.70 ± 0.22	0.41 ± 0.21
3 uM LD	2.32 ± 1.06	3.36 ± 1.20	1.88 ± 1.97	0.64 ± 0.22	0.29 ± 0.08
5 uM LD	3.50 ± 1.36	3.62 ± 0.33	0.92 ± 1.50	0.70 ± 0.15	0.36 ± 0.16
34-37-40 °C	2.76 ± 0.78	3.90 ± 0.82	1.27 ± 1.43	0.70 ± 0.18	0.29 ± 0.13

Table M2: **Parameters associated with positive region of F_1.** 37 °C A refers to the 37 °C data that were fitted alone, while 34-37-40 °C refers to the three temperatures that were fitted together. K_1^+ is the mean coupling constant that multiply positive Gaussians in F_1, while μ^+ and Σ^+ are the means and the standard deviations of the Gaussians, in the two coordinates θ and ϕ.

Condition	$K_1^-[rad \times h^{-1}]$	$\mu_\theta^-[rad]$	$\mu_\phi^-[rad]$	$\Sigma_\theta^-[rad]$	$\Sigma_\phi^-[rad]$
37 °C A	-1.28 ± 0.95	3.39 ± 1.91	3.32 ± 1.94	0.91 ± 0.72	0.36 ± 0.51
1 uM LD	-2.35 ± 0.96	3.30 ± 1.03	2.22 ± 1.33	0.57 ± 0.21	0.40 ± 0.17
3 uM LD	-1.45 ± 1.17	3.85 ± 2.32	4.20 ± 1.75	0.51 ± 0.22	0.34 ± 0.11
5 uM LD	-1.65 ± 1.12	3.20 ± 1.50	2.09 ± 1.51	0.66 ± 0.27	0.33 ± 0.18
34-37-40 °C	-1.99 ± 1.05	3.25 ± 1.43	3.12 ± 0.96	0.77 ± 0.26	0.31 ± 0.13

Table M3: **Parameters associated with negative region of F_1.** 37 °C A refers to the 37 °C data that were fitted alone, while 34-37-40 °C refers to the three temperatures that were fitted together.

Condition	$K_2^+[rad \times h^{-1}]$	$\mu_\theta^+[rad]$	$\mu_\phi^+[rad]$	$\Sigma_\theta^+[rad]$	$\Sigma_\phi^+[rad]$
37 °C A	1.32 ± 2.40	2.56 ± 1.61	2.35 ± 2.08	0.66 ± 0.60	0.57 ± 0.51
1 uM LD	0.99 ± 0.73	3.94 ± 1.46	2.35 ± 1.70	0.57 ± 0.26	0.43 ± 0.24
3 uM LD	0.84 ± 0.49	3.00 ± 1.05	1.71 ± 2.02	0.42 ± 0.14	0.38 ± 0.17
5 uM LD	0.94 ± 1.02	2.52 ± 1.49	2.64 ± 2.09	0.50 ± 0.25	0.39 ± 0.13
34-37-40 °C	1.48 ± 1.27	3.89 ± 0.43	2.27 ± 1.33	0.39 ± 0.17	0.42 ± 0.14

Table M4: **Parameters associated with position region of F_2.** 37 °C A refers to the 37 °C data that were fitted alone, while 34-37-40 °C refers to the three temperatures that were fitted together.

Condition	$K_2^- [rad \times h^{-1}]$	$\mu_\theta^- [rad]$	$\mu_\phi^- [rad]$	$\Sigma_\theta^- [rad]$	$\Sigma_\phi^- [rad]$
37 °C A	-1.32 ± 2.95	4.34 ± 1.47	3.42 ± 1.51	0.62 ± 0.52	0.81 ± 0.62
1 uM LD	-0.57 ± 0.33	1.16 ± 0.94	2.77 ± 1.61	0.64 ± 0.22	0.63 ± 0.29
3 uM LD	-0.62 ± 0.55	2.44 ± 1.60	2.76 ± 1.17	0.46 ± 0.20	0.52 ± 0.23
5 uM LD	-0.58 ± 0.21	3.22 ± 1.99	3.76 ± 1.62	0.42 ± 0.21	0.51 ± 0.23
34-37-40 °C	-1.07 ± 0.65	3.30 ± 1.84	3.30 ± 1.52	0.49 ± 0.29	0.63 ± 0.32

Table M5: **Parameters associated with negative region of F_2.** 37 °C A refers to the 37 °C data that were fitted alone, while 34-37-40 °C refers to the three temperatures that were fitted together.

E. Validation and parameters identifiability

In order to validate our fitting procedure we generated data from our model using realistic parameters ($T_1 = 24$h, $T_2 = 22$h,$\sigma_1 = 0.15$, $\sigma_1 = 0.2$) and unidirectional coupling functions. We then inferred the parameters, the resulting coupling functions are shown in Figure M9 and Figure M10.

Our fitting procedure is able to predict unambiguously the directionality of the coupling (almost no interactions are predicted in Figure M9C and Figure M9A). It can also predict the position of the interaction relatively precisely, even though there is clearly some soft indeterminacy in the diagonal direction (the interactions are spread along the diagonal in Figure M10C). The deceleration in Figure M9B seems also hard to recover, though this might be due to the fact that this interaction is just behind the other one, in the diagonal direction, and thus might have effectively little impact on the dynamic of the trajectories. The stable manifolds are well predicted (Figure M9E and Figure M10E).

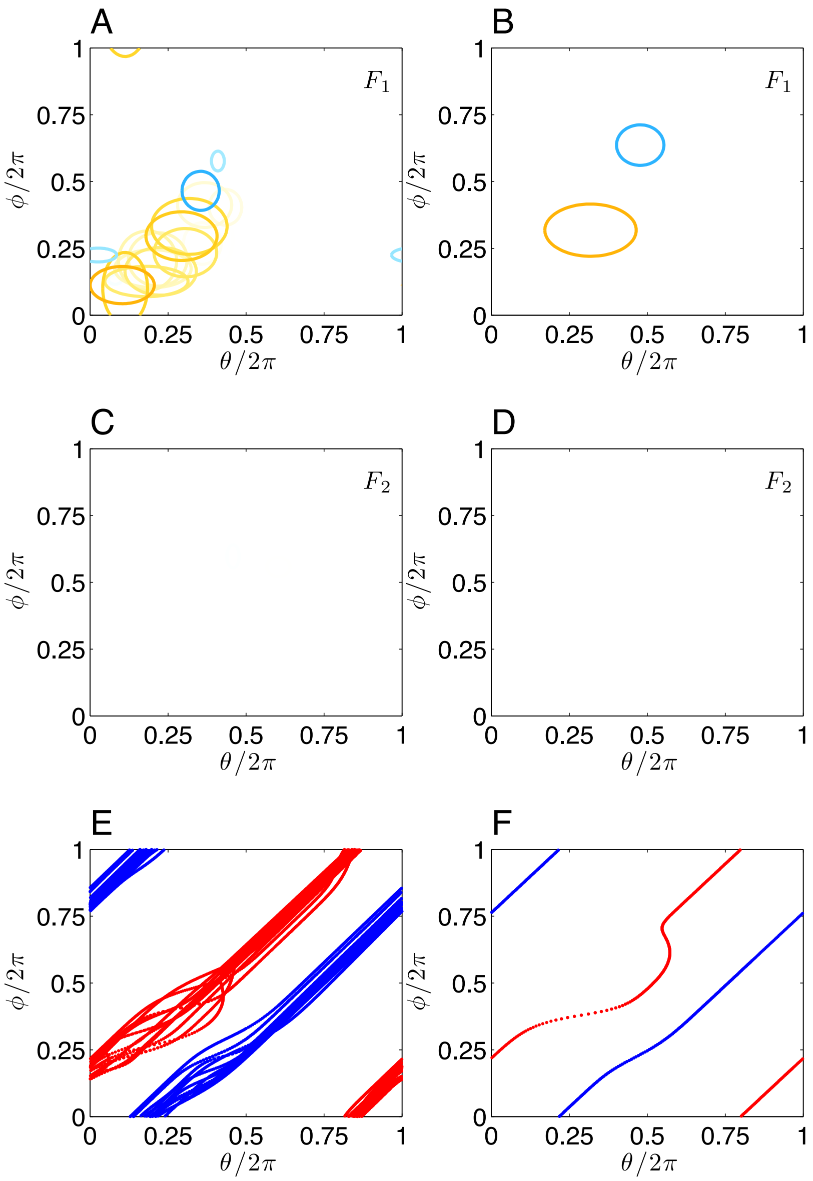

Figure M9: **Validation of model inference** The model was fitted on simulated data with $F_2 = 0$ and F_1 as shown in B ($K_1 = 2.9$, $K_2 = -1.2$). **A.** Inferred coupling function F_1. Orange regions indicate significant acceleration of the circadian phase by the cell cycle, while blue regions indicate a deceleration of the circadian phase. **B.** Coupling function F_1 used to generate the data. **C.** Inferred coupling function F_2. **D.** Coupling function F_2 used to generate the data. **E.** Stable (blue) and unstable (red) manifolds in the fitted models. **F.** Stable (blue) and unstable (red) manifolds in the model used to generate the data.

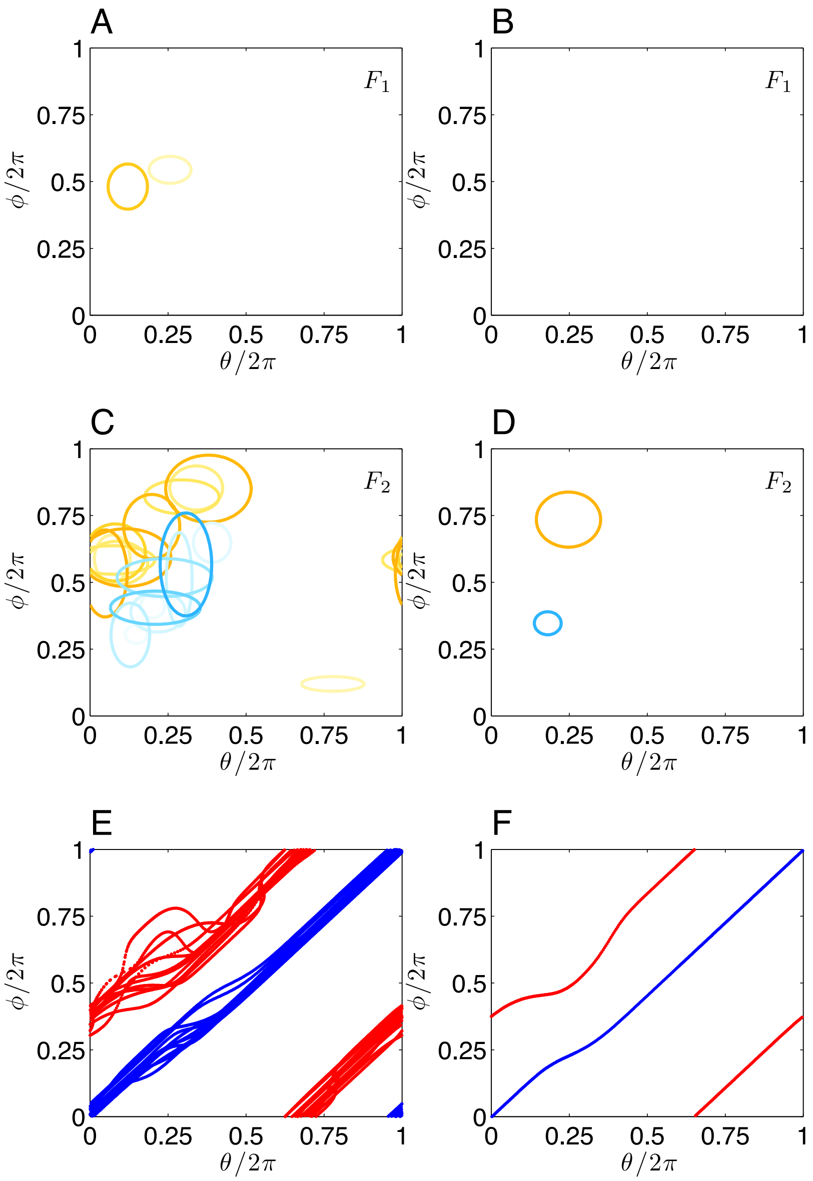

Figure M10: **Validation of model inference** The model was fitted on simulated data with $F_1 = 0$ and F_2 as shown in B ($K_3 = 2.9$, $K_4 = -1.2$). **A.** Inferred coupling function F_1. **B.** Coupling function F_1 used to generate the data. **C.** Inferred coupling function F_2. Orange regions indicate significant acceleration of the cell cycle phase by the circadian clock, while blue regions indicate a deceleration of the cell cycle phase. **D.** Coupling function F_2 used to generate the data. **E.** Stable (blue) and unstable (red) manifolds in the fitted models. **F.** Stable (blue) and unstable (red) manifolds in the model used to generate the data.

III. PHASE INFERENCE USING A HIDDEN MARKOV MODEL

In most analyses we used circadian peak times, corresponding to local maxima of the circadian signal as the time when the circadian phase goes through 2π. However the complete circadian signal contains additional information about the phase progression through the circadian cycle. We used a hidden Markov model (HMM) to infer the phase of the circadian cycle from our recordings.

In addition to the circadian phase, we also need to assume a time varying amplitude as the amplitude of the signal in our data can vary from peak to peak.

The model links the observed circadian signal $s(t)$ to the circadian phase $\theta(t)$ through a waveform $w(\theta)$ and an amplitude $A(t)$:

$$s(t) = A(t)w(\theta(t)) + \xi, \tag{4}$$

where ξ is normally distributed random variable with zero mean. For the waveform we used the function $w(\theta) = (1/2 + 1/2\cos(\theta))^{\alpha}$ with $\alpha = 1.6$. This simple waveform corresponds reasonably well to the data.

As in our stochastic phase model (Section II), the phase variable is modeled by a diffusion-drift equation, i.e. the phase increments $\theta(t+\Delta t) - \theta(t)$ are normally distributed with mean $\frac{2\pi}{T}\Delta t$ and variance $\sigma^2\Delta t$, where T is the circadian period and σ the circadian phase diffusion coefficient:

$$\mathrm{d}\theta_t = \frac{2\pi}{T}\mathrm{d}t + \sigma \mathrm{d}W_t \tag{5}$$

We used a mean period of 24h and a diffusion coefficient of 0.15, corresponding to a standard deviation on the circadian intervals of 2.8h and consistently with the values found in our stochastic phase model.

The amplitude $A(t)$ is modelled by $\exp(\lambda(t))$ where $\lambda(t)$ is an Ornstein-Uhlenbeck process with zero mean :

$$\mathrm{d}\lambda_t = -\gamma\lambda_t \mathrm{d}t + \sigma_\lambda \mathrm{d}W_t \tag{6}$$

An Ornstein-Uhlenbeck process converges on average to its mean, thus the amplitude $A(t)$ will stay close to unity on average, but is able to fluctuate to compensate for large amplitude

changes in the data. The parameter γ defines the time scale with which the process relaxes to its mean, here we used $1/\gamma = 24$h and $\sigma_\lambda = 0.08$ which gives a standard deviation of the amplitude $A(t)$ of ~ 0.3 around the mean of 1.

The circadian signal was preprocessed by normalizing its amplitude and removing its linear trend. The dip in the signal during the division was also masked to avoid spurious distortion of the phase at mitosis.

Using the standard framework of HMMs [1] in which the joined probability of signal s and hidden states θ and λ factors as $P(s, \theta, \lambda) = P_e(s|\theta, \lambda)Q(\theta, \lambda)$ where P_e is the total emission probability, we computed the most likely sequence of phases and amplitudes for each trace using the Viterbi algorithm.

The emission term was computed using Equation 4 and the probability Q was obtained from solving propagators (transition probabilities) corresponding to Equations 5 and 6.

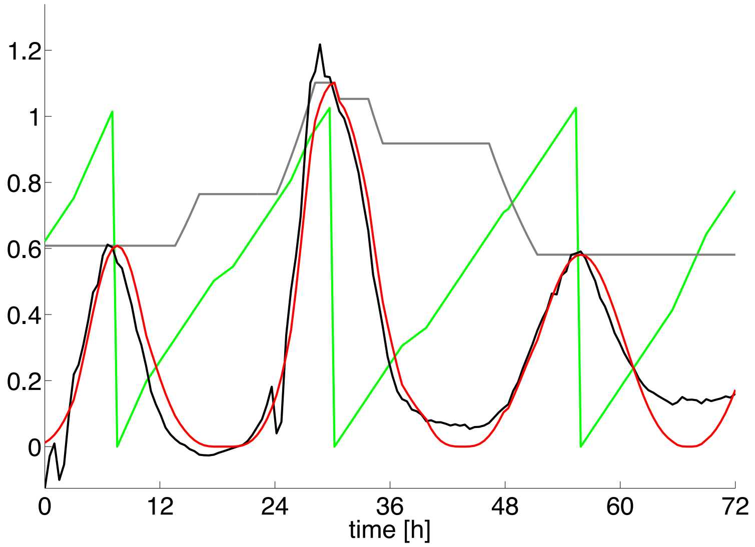

Figure M11: **Example of inferred phase and amplitude.** Black: Circadian trace at 37 °C. Red: inferred signal. Green: inferred phase. Gray: inferred amplitude.

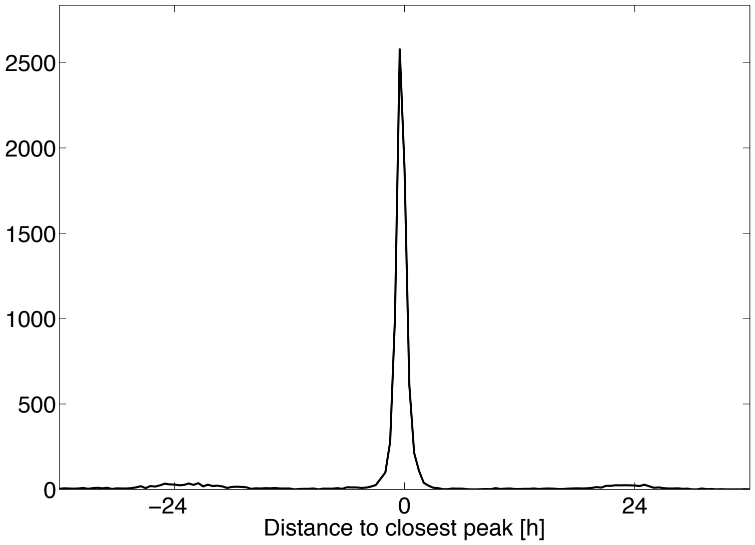

Figure M12: **The HMM recovers the manually validated circadian peak timings.** Distribution of distance from a manually validated peak to the closest peak found by the HMM. Only a small fraction of peaks are not found as indicated by the tiny bumps around -24 and 24h.

A. Validation

In order to validate our inference procedure we simulated data using our stochastic phase model, using reasonable noise values and coupling constants, and inferred back the phase and amplitude using our HMM. Two additional ingredients were necessary to generate realistic signals at shown in Figure M13: a waveform which we took as $w(\theta) = (1/2 + 1/2\cos(\theta))^\alpha$ with $\alpha = 1.3$ and an amplitude that we generated by simulating an Ornstein-Uhlenbeck process as described above.

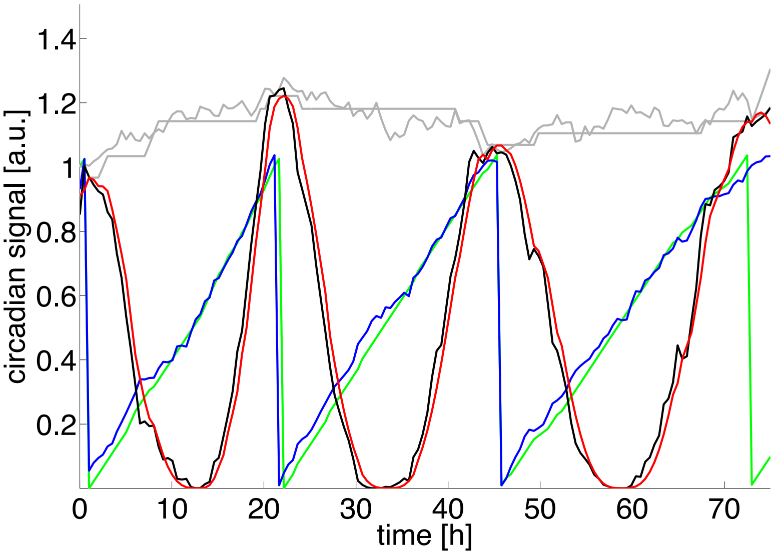

Figure M13: **Simulated signal used for the validation.** Black: Simulated signal. Red: inferred signal. Blue: simulated phase. Green: inferred phase. Gray: simulated and inferred amplitude.

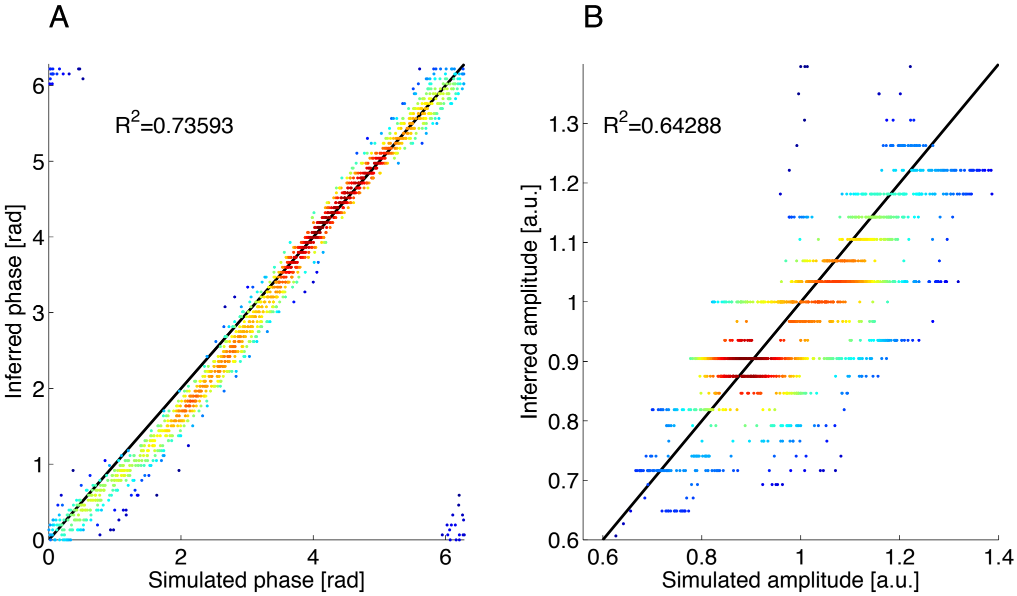

Figure M14: **The HMM infers accurately the phase of the simulated oscillator. A.** The correlation between the simulated phase and the inferred phase is very good. The slight deviation from the diagonal indicates that the waveform used in the model and in the inference procedure is not exactly the same ($\alpha = 1.3$ vs 1.6). The color scale corresponds to the data point density. **B.** The amplitude is well predicted.

IV. SYNCHRONIZATION INDICES

In order to quantify the synchrony of the circadian phase across cells we computed the order parameter R_θ defined by:

$$R_\theta(t)\exp[i\Psi_\theta(t)] = \frac{1}{N}\sum_{k=1}^{N}\exp[i\theta_k(t)]. \tag{7}$$

Where θ_k is the circadian phase of cell k. The order parameter R_θ takes values between zero (no synchrony: the phases are uniformly distributed along the unit circle in the complex plane) and one (complete synchrony). Note that when N is finite, R_θ does not reach exactly zero values for unsynchronized phases (finite size effect, cf. Figure 7). We define the order parameter for the cell cycle (R_ϕ) in a similar way. The synchrony between the cell cycle and the circadian clock ($R_{\theta,\phi}$) is measured by the coherence of phase differences across cells:

$$R_{\theta,\phi}(t)\exp[i\Psi_{\theta,\phi}(t)] = \frac{1}{N}\sum_{k=1}^{N}\exp[i\theta_k(t) - i\phi_k(t)]. \tag{8}$$

Here, we estimated θ_k by assuming that the circadian phase goes linearly from 0 to 2π between two observed peaks. In order to quantify the error on our order parameters we estimated the variance of $\theta_k(t)$ as follows. Because we observe only finite traces we have to deal with two different cases: first we need to estimate the phase at the end (or beginning) of a trace, in which only one peak is available on the left (or right) of the trace. Secondly, we need to estimate the phase between two peaks. In the first case, assuming the phase follows a Brownian motion with drift (Eq. 5), the phase at time t is given by a normal distribution with mean $2\pi/T_1(t - p_M)$ and variance $(t - p_M)\sigma_1$, where p_M is the time of the last peak of the trace, T_1 and σ_1 are the intrinsic period and the the noise parameter of the circadian phase. For the phase at the beginning of a trace we use the same distribution with reversed time.In the second case the phase at time t is given by a normal distribution with mean $2\pi(t - p_1)/(p_2 - p_1)$ and variance $(t - p_1)(p_2 - t)/(p_2 - p_1)\sigma_1$ where p_1 and p_2 are the times of the two peaks encompassing the circadian phase. This distribution is the solution to the diffusion-drift equation with Dirac boundary conditions at p_1 and p_2. Figure M15 shows these quantities for a trace with two peaks.

An analogous procedure is done for the cell cycle phase $\phi_k(t)$. Finally, the means and variances of the order parameters R_θ, R_ϕ and $R_{\theta,\phi}$ are computed by sampling these phase distributions.

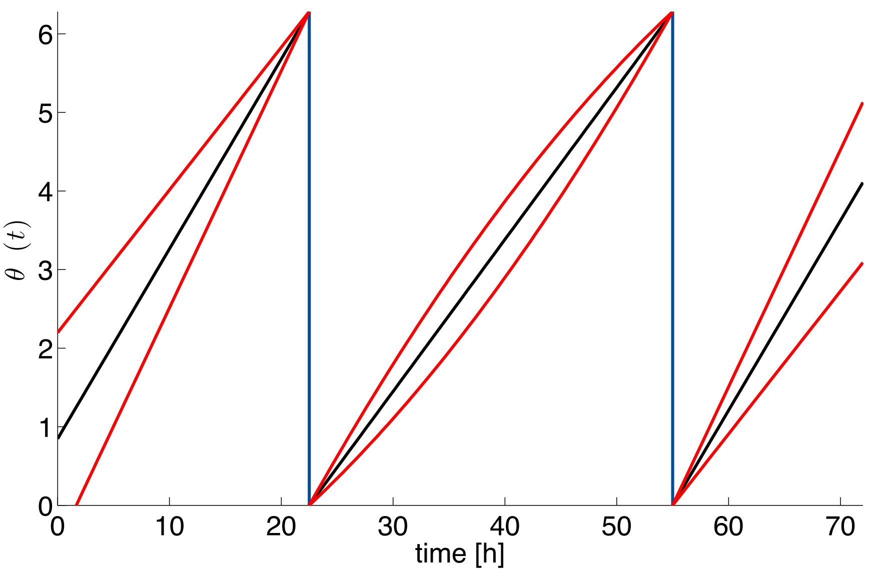

Figure M15: **Illustration of phase estimation.** The mean (black) and variance (red) of the estimated circadian phase between two peaks (blue) is shown. Note that the phase is more constrained between two peaks than at the boundaries.

V. GRANGER CAUSALITY

A. Nuclear area and cell cycle phase

In addition to the fluorescent signal we also measure the area of the nucleus from our segmentation and tracking analysis. It is known [2] that the nucleus volume increases during the cell cycle in mammalian cells. We thus hypothesized that the nucleus area contains information about the cell cycle progression. To test this we first plotted the nuclear area for a few cells, as shown in Figure M16 the nucleus area increases between two divisions with a characteristic pattern, before decreasing by about a factor of two at mitosis. The nuclei seem to regrow rapidly for about 4 to 5 hours after the division and then increase in size at slower pace. This is clearly visible when looking at the nucleus area averaged over many cell cycles (Figure M17), where we find a pattern that is very similar that reported in [2]. We verified that the rapid increase after a division is not due to movement in the z-axis, as nuclei regain focus after about one hour following division in our recordings.

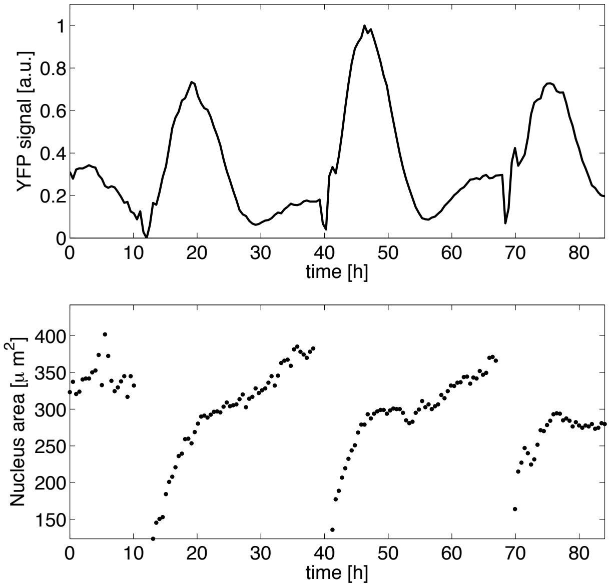

Figure M16: **Circadian signal and nucleus area**

The normalized circadian signal is shown on top, and the nucleus area on the bottom. The area in μm^2 was computed from the objective magnification (20x) and the camera pixel size (16x16 μm). This gives nuclei radii around 5 to 10 μm.

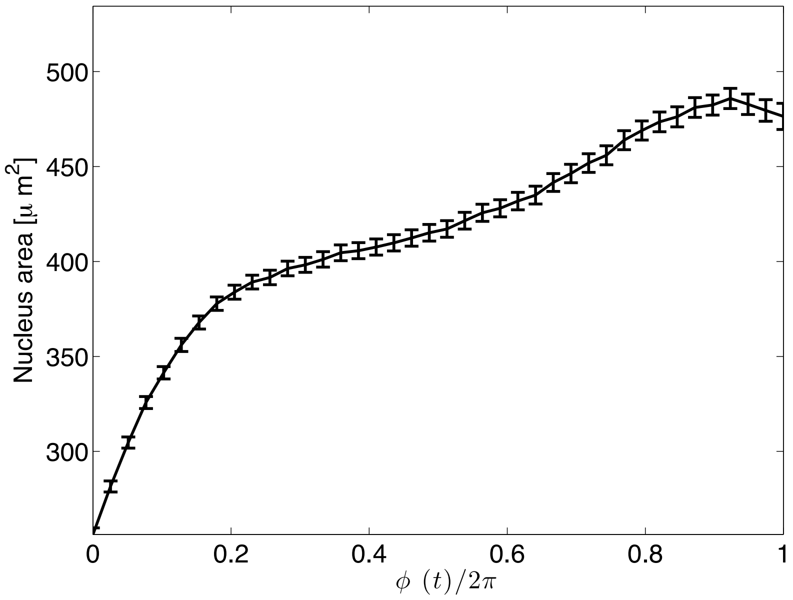

Figure M17: **Mean nucleus area during the cell cycle** The mean (n=836) nucleus area is shown in function of the cell cycle phase. Here we assumed that the cell cycle phase goes linearly from 0 to 2π between two mitosis. The error bars represent the standard error on the mean.

To confirm that nuclear area contains information about the state of the cell cycle we analyzed a previously publish movie of HeLa cells containing the FUCCI system (movie S1 in [7]). After segmentation of the movie this allowed us to estimate the time of onset of S phase in 31 cells. The estimated S phase onset is shown in a single cell in Figure M18 and the average S phase onset is shown alongside the mean nucleus area in Figure M19.
We then aimed at predicting the measured times of S phase onset, denoted t_S, by using only the normalized nucleus area $n(t)$. For a given cell we computed the probability that time t is t_S as:

$$P(t = t_S) = \mathrm{G}(n(t), \dot{n}(t); \mu, \Sigma)$$

Where G is a bivariate Gaussian with mean μ and covariance matrix Σ, while $\dot{n}(t)$ is the time derivative of $n(t)$. We split our data into a training set and a test set, and estimated μ and Σ from the training set. As shown in Figure M20 this simple model is able to predict the onset of S phase with good accuracy, demonstrating that the nuclear size does contain information on the cell cycle progression.

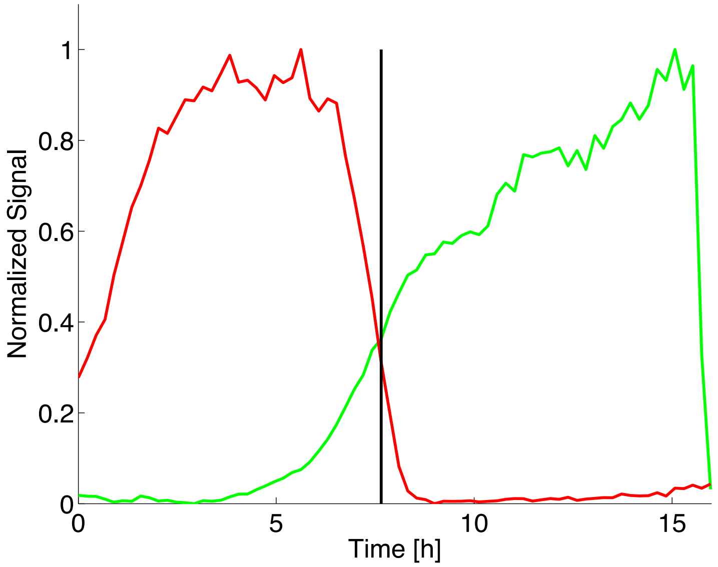

Figure M18: **Measuring S phase onset in HeLa cells.** Red : Measured mKO2-hCdt1 signal in a single cell between two divisions. Green: Measured mAG-hGem signal. The vertical black line represent the estimated S phase (time of crossing of red and green signal).

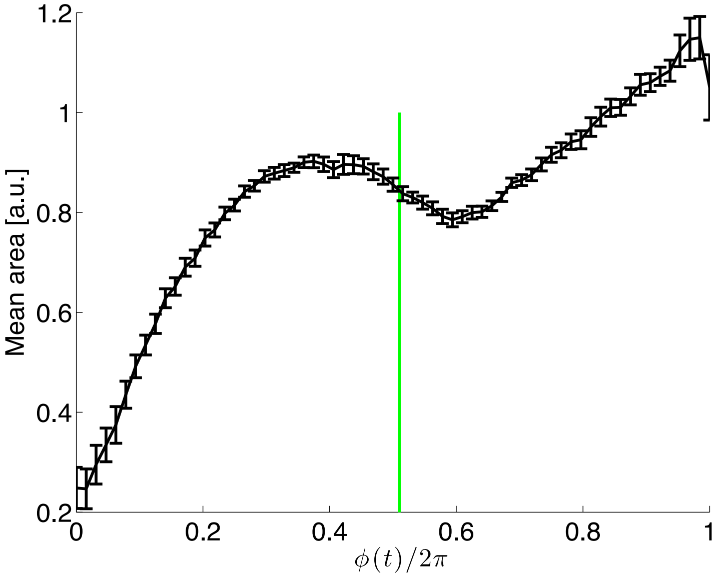

Figure M19: **Mean S phase onset and mean nucleus area in HeLa cells.** The mean normalized nucleus area is shown in black (error bars represent the standard error on the mean) against the cell cycle phase. The mean estimated phase of S phase onset (0.51 ± 0.07) is shown in green.

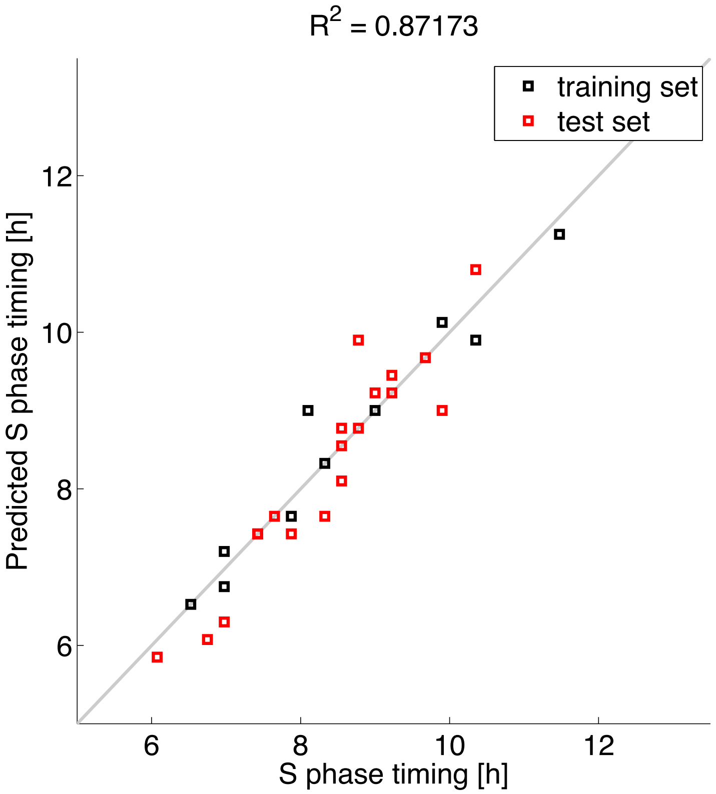

Figure M20: **Nucleus area predicts S phase onset in HeLa cells**

B. Granger Causality

We applied the Granger-Wald causality test as defined in [5] to each trace (we used only traces longer than 60 hours) independently. For a given lag L in the autoregressive model, we applied the test directly to the raw circadian signal and nucleus area, and decided for each trace if the circadian signal was Granger-causing the nucleus area or vice-versa using the Granger-Wald test with a p-value of 10^{-3}. Finally we counted the fraction of traces falling in each category. The results are shown in Figure S8.

[1] Richard Durbin, Sean R. Eddy, Anders Krogh, and Graeme Mitchison. *Biological Sequence Analysis: Probabilistic Models of Proteins and Nucleic Acids.* Cambridge University Press, 1998.

[2] J Fidorra, T Mielke, J Booz, and L E Feinendegen. Cellular and nuclear volume of human cells during the cell cycle. *Radiation and environmental biophysics*, 19(3):205–14, January 1981.

[3] J. L. Folks and R. S. Chhikara. The inverse gaussian distribution and its statistical application–a review. *Journal of the Royal Statistical Society. Series B (Methodological)*, 40(3):pp. 263–289, 1978.

[4] N. Hansen and A. Ostermeier. Completely derandomized self-adaptation in evolution strategies. *Evol Comput*, 9(2):159–95, 2001.

[5] K Hlavackova-Schindler, M Palus, M Vejmelka, and J Bhattacharya. Causality detection based on information-theoretic approaches in time series analysis. *Physics Reports*, 441(1):1–46, March 2007.

[6] Khuloud Jaqaman, Dinah Loerke, Marcel Mettlen, Hirotaka Kuwata, Sergio Grinstein, Sandra L Schmid, and Gaudenz Danuser. Robust single-particle tracking in live-cell time-lapse sequences. *Nature methods*, 5(8):695–702, August 2008.

[7] Asako Sakaue-Sawano, Hiroshi Kurokawa, Toshifumi Morimura, Aki Hanyu, Hiroshi Hama, Hatsuki Osawa, Saori Kashiwagi, Kiyoko Fukami, Takaki Miyata, Hiroyuki Miyoshi, Takeshi Imamura, Masaharu Ogawa, Hisao Masai, and Atsushi Miyawaki. Visualizing spatiotemporal dynamics of multicellular cell-cycle progression. *Cell*, 132(3):487–98, March 2008.

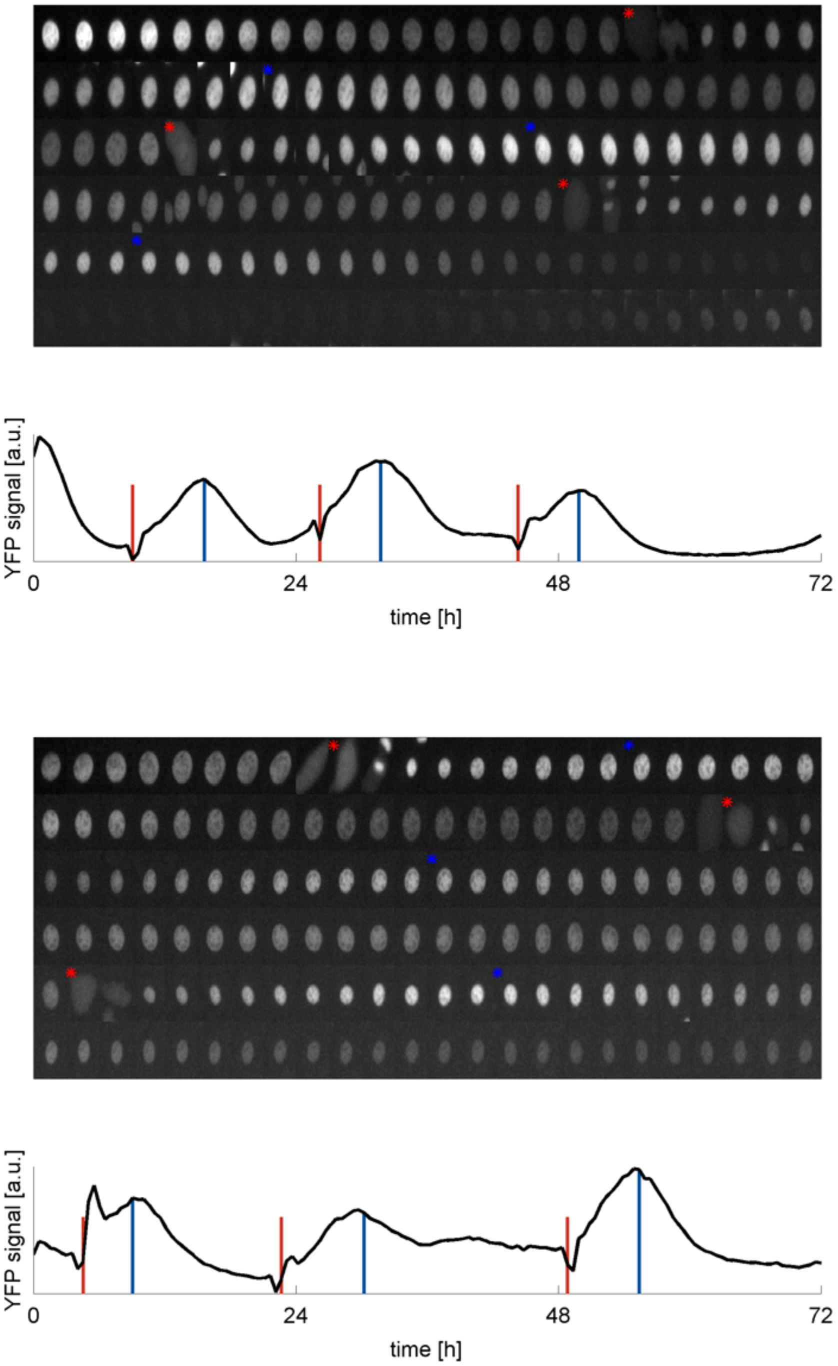

Figure S1. Time series of circadian *Rev-Erb*α-YFP signals in tracked nuclei.

Two time series of tracked nuclei are shown along with YFP signals underneath. Time goes from top left to bottom right, each of the six rows represent 12 hours. Images are taken every 30 minutes for a total of 72 hours. Stars indicate frames with circadian peaks (blue) or divisions (red). Below the images, the circadian signal (black) is shown with detected peaks (blue) and divisions (red).

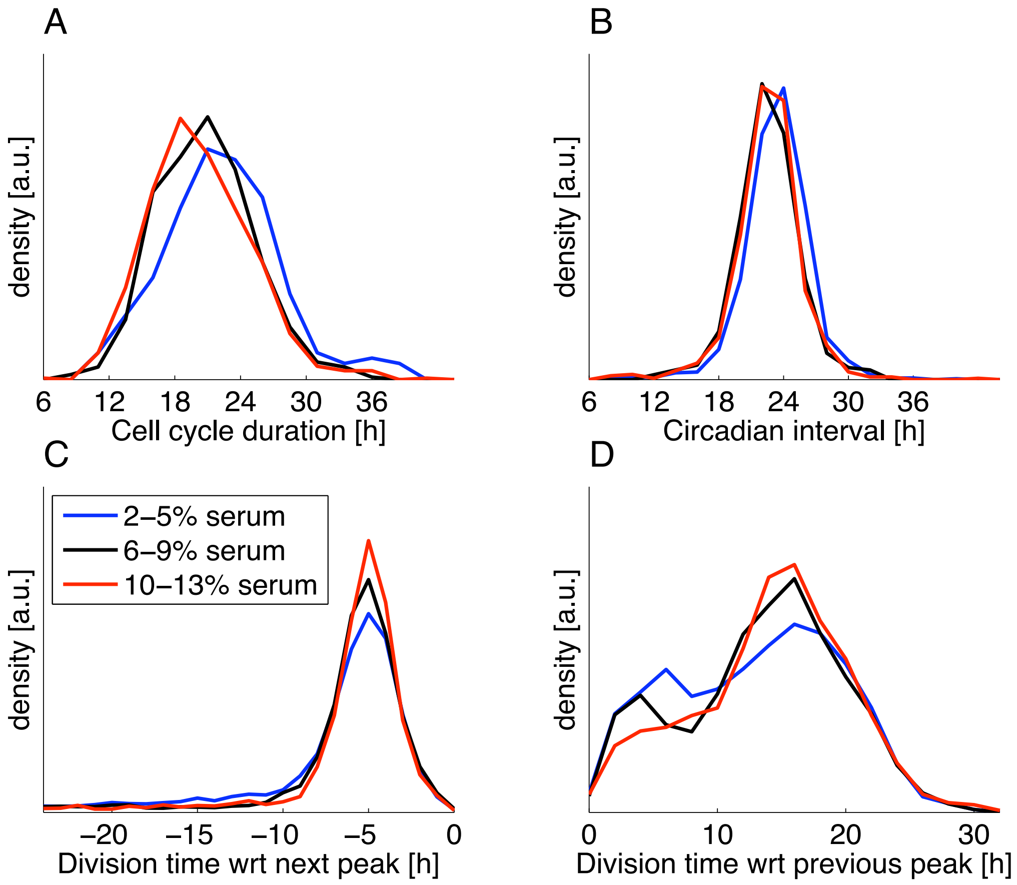

Figure S2. Effect of serum concentration on circadian intervals and cell cycle durations.

A. The distribution of cell-cycle duration shows a small dependence on serum concentration (the distribution is shown for 2-5%, 6-9% and 10-13% serum). B. Distribution of circadian intervals does not vary much with serum concentration. C. The distribution of division time is similar for all serum concentrations. D. The distribution of division time w.r.t. previous circadian peak shows a minor dependence on serum concentration. The number of traces analyzed here were n=1465, n=1288 and n=3967 in the low, medium and high concentration range, respectively.

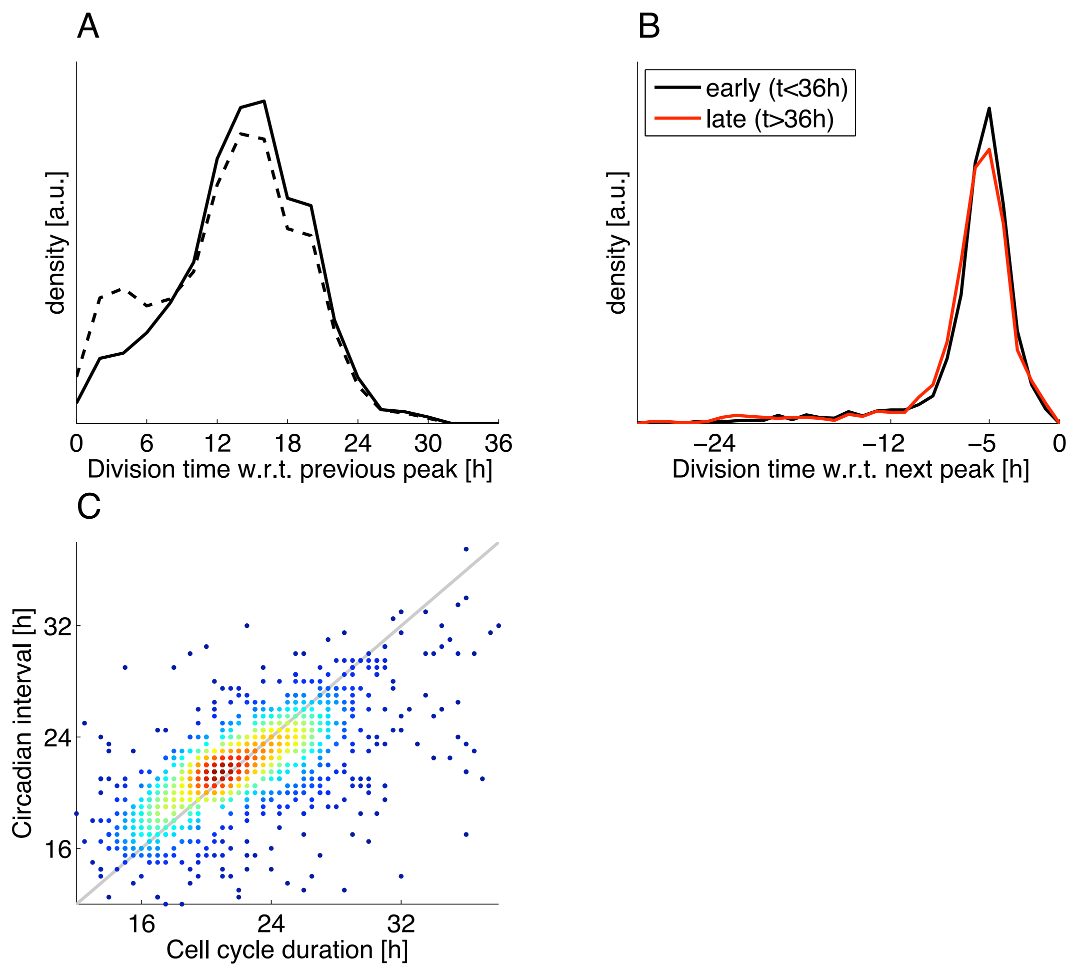

Figure S3. Circadian and cell cycle oscillators are tightly synchronized at 37 ºC.

A. The interval length from the previous circadian peak to the division time (p,d) is less constrained than (d,p) interval length (Figure 1C). Solid line: histogram of (p_1,d_1) subintervals from (p_1,d_1,p_2) events; dashed line: all (p,d) intervals. The population of short (p,d) intervals is reduced among (p_1,d_1,p_2) events. B. The distribution of (d,p) intervals is homogenous in time: events in the first half (black) of the recordings are no different than during the second half (red). C. Circadian intervals (peak-to-peak times) and cell cycle durations are tightly correlated. Color indicates density of measurements.

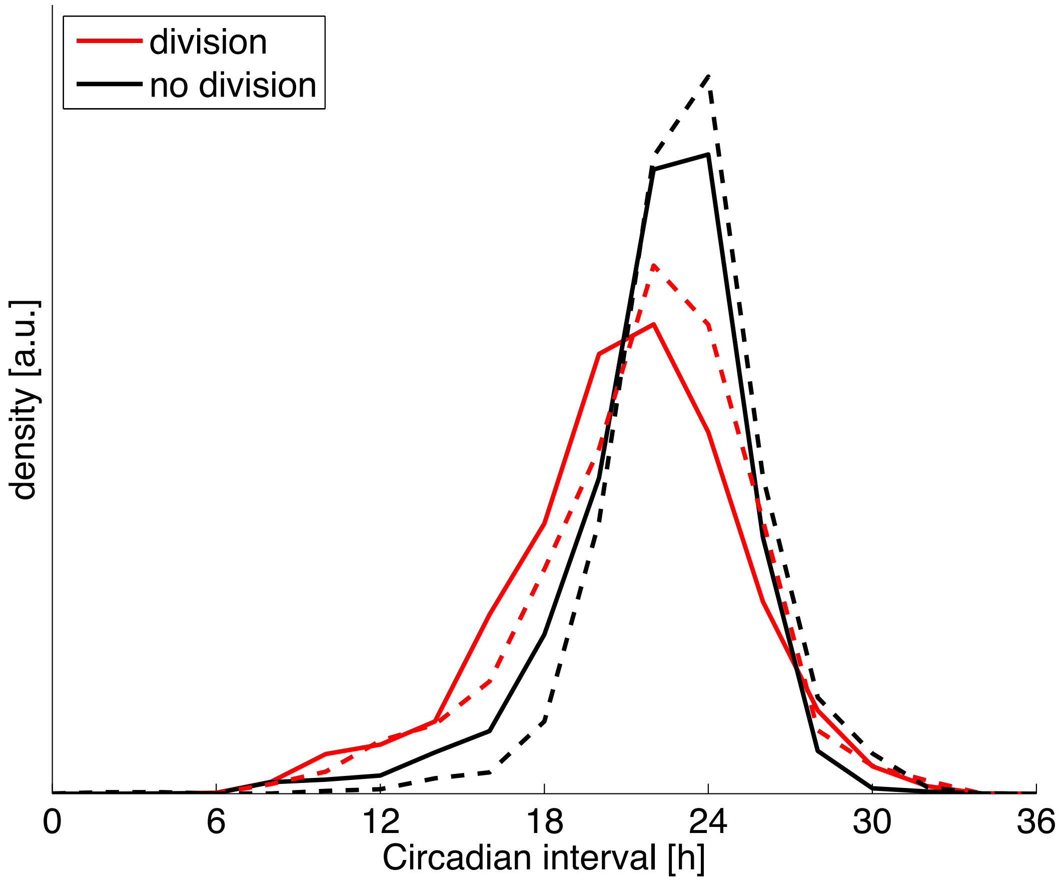

Figure S4. The influence of cell-cycle time on circadian phase progression is homogenous in time. Circadian intervals with (black) and without (red) divisions in the first (solid) and second (dashed) halves of the recordings show significant shifts (first half, $p<10^{-7}$, t-test; second half, $p<10^{-9}$, t-test).

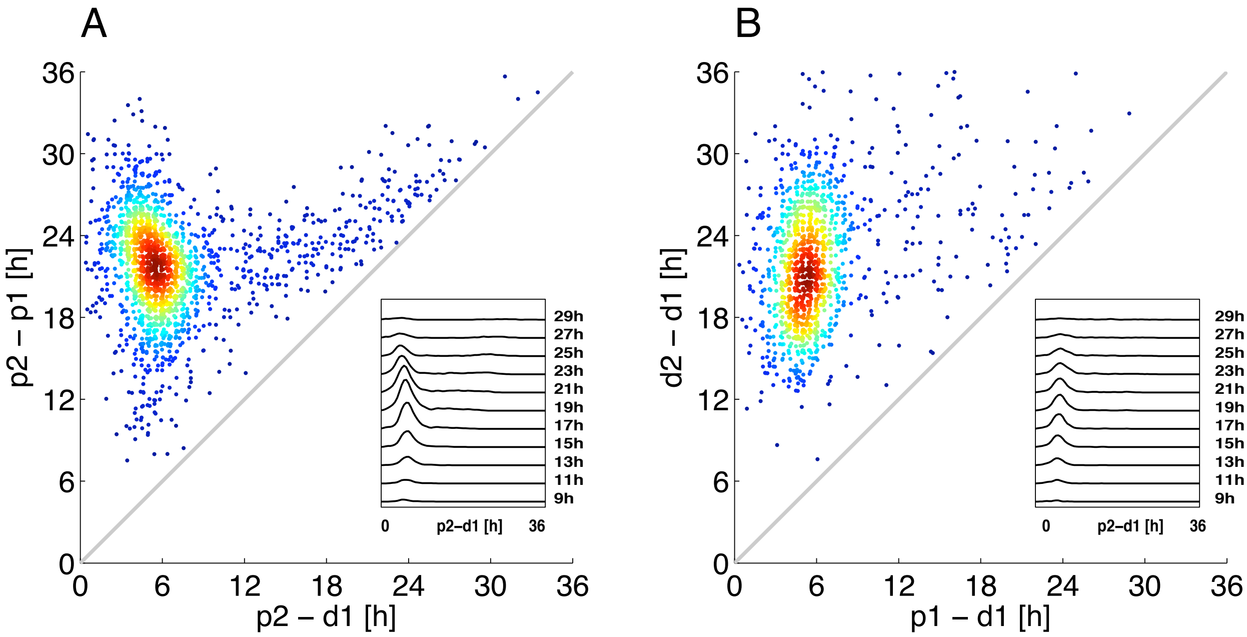

Figure S5. Signature of unidirectional coupling from the cell cycle onto the circadian phase for normally growing cells at 37 ºC.

A. Full circadian intervals (p_1,p_2) in (p_1,d_1,p_2) events compared to the second subinterval (d_1,p_2) shows early division events as outliers along the diagonal. The negative correlation of (p_1,p_2) vs. (d_1,p_2) in the center of the distribution coincides with the positive correlation centered on phases ~0.8 in Figure 2B and is a property of the fitted stochastic model in which the influence of the cell cycle onto the circadian oscillator dominates (cf. Figure 3). Insets show the marginal distributions (times on the x-axis stratified according to the y-axis), shifting towards smaller times. B. Similar representation for (d_1,p_1,d_2) to probe the reverse interaction of the circadian cycle onto the cell cycle. Unlike A), the distribution shows a positive correlation that is consistent with a proportional stretching of the (d_1,p_1) subintervals as the enclosing (d_1,d_2) duration lengthens (see main text).

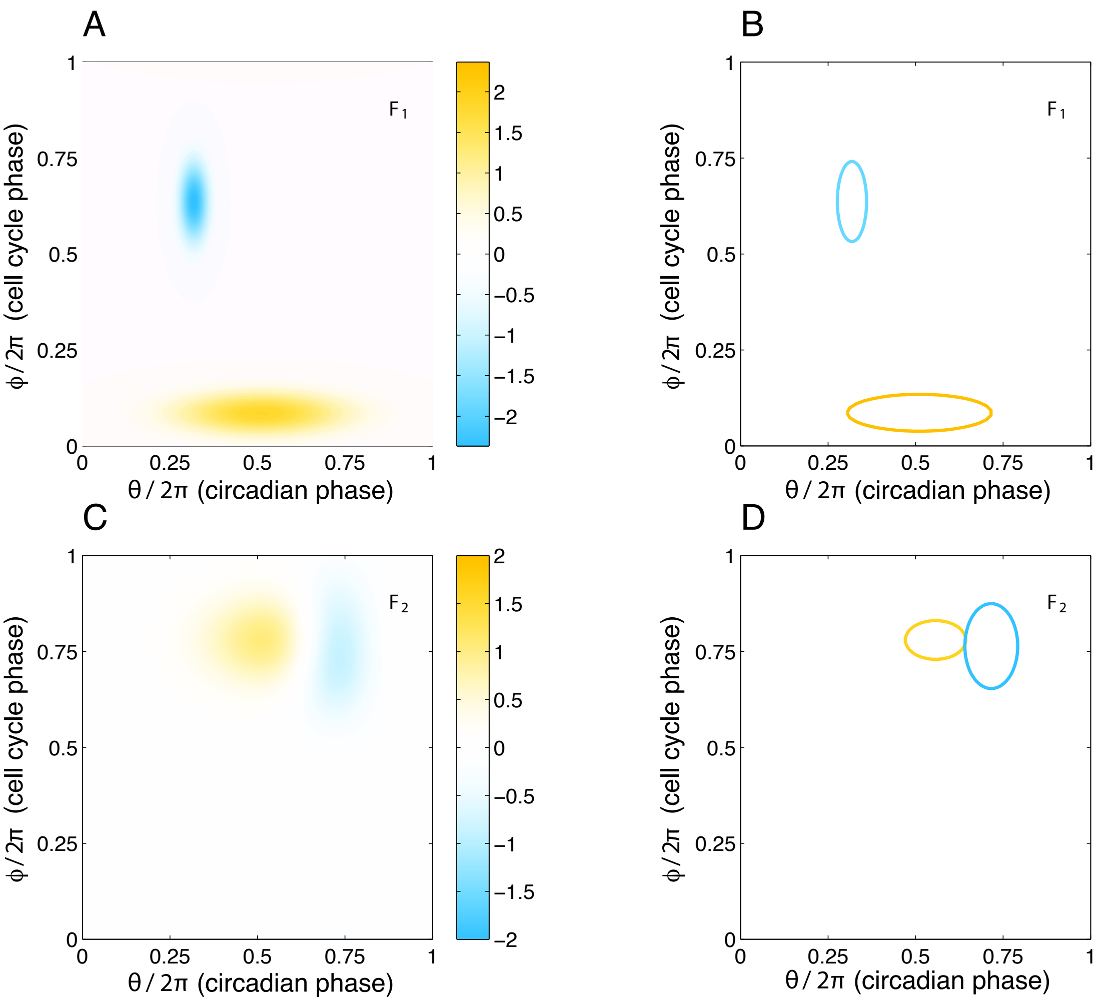

Figure S6. Representation of coupling functions in the generic model used to fit the data.

A-B. The influence of the cell-cycle on the circadian clock can be described by two Gaussian functions arbitrarily placed and sized, which can either accelerate (yellow) or decelerate (blue) the circadian phase progression. Covariance is diagonal in the coordinates used. A: the density of the elliptical function; B: the contours correspond to a fixed (absolute) value of the function, as shown in the main figures. C-D. Idem for the coupling functions describing the influence of the circadian cycle on cell division. The functions shown here were chosen arbitrarily for illustration purpose.

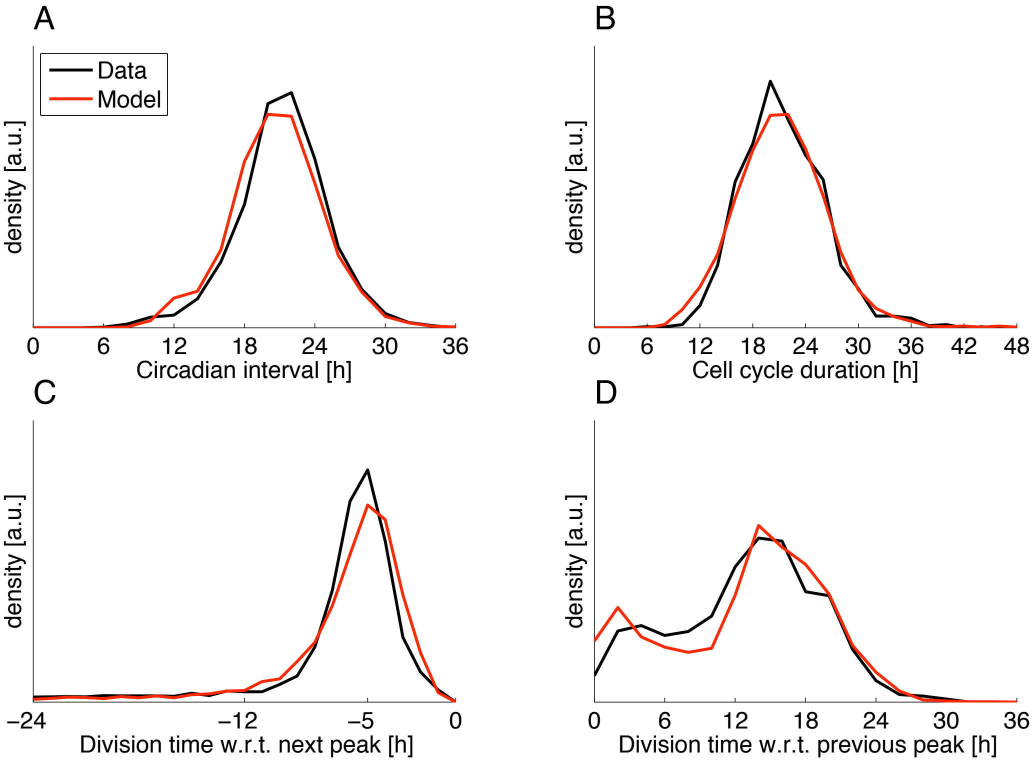

Figure S7. Comparison of fit and data at 37 ºC for the best-fit model.

A. The distributions of circadian intervals (p_1,d_1,p_2).

B. The distributions of cell-cycle durations (d_1,p_1,d_2).

C. The interval from divisions to the next circadian peaks (d,p).

D. The interval between the previous peaks and the divisions (p,d).

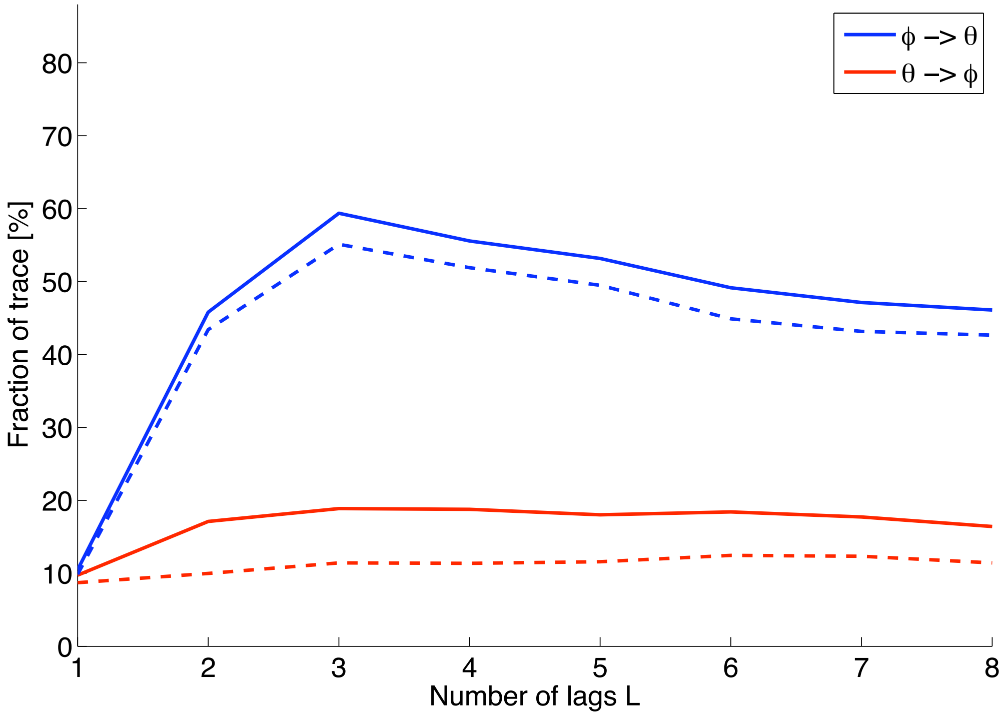

Figure S8. Testing for Granger causality. Proportion of cells showing significant evidence ($p<0.001$, Granger-Wald test) that cell cycle progression (assessed via the nuclear size, Supplementary Information section VA) predicts the circadian *Rev-Erbα*–YFP signal (solid blue), or vice versa (solid red), in function of the number of lags used in the autoregressive models. Dashed lines show the proportion of cells for which the detected causality ($p<0.001$) is more significant than that of the reverse direction.

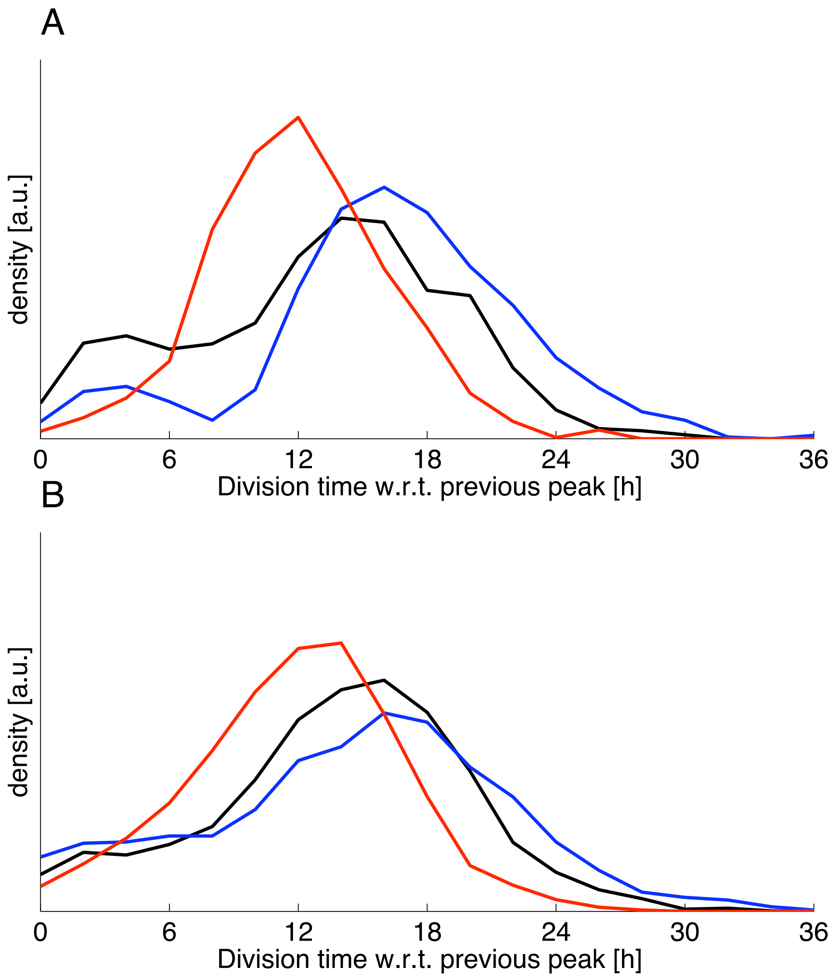

Figure S9. Distributions of division times measured with respect to the previous circadian peak in temperature experiments.

A. Division times measured with respect to the previous circadian peak are strongly affected by temperature (blue: 34 ºC, black 37 ºC, red: 40 ºC). B. Division times measured with respect to the previous circadian peaks in the model.

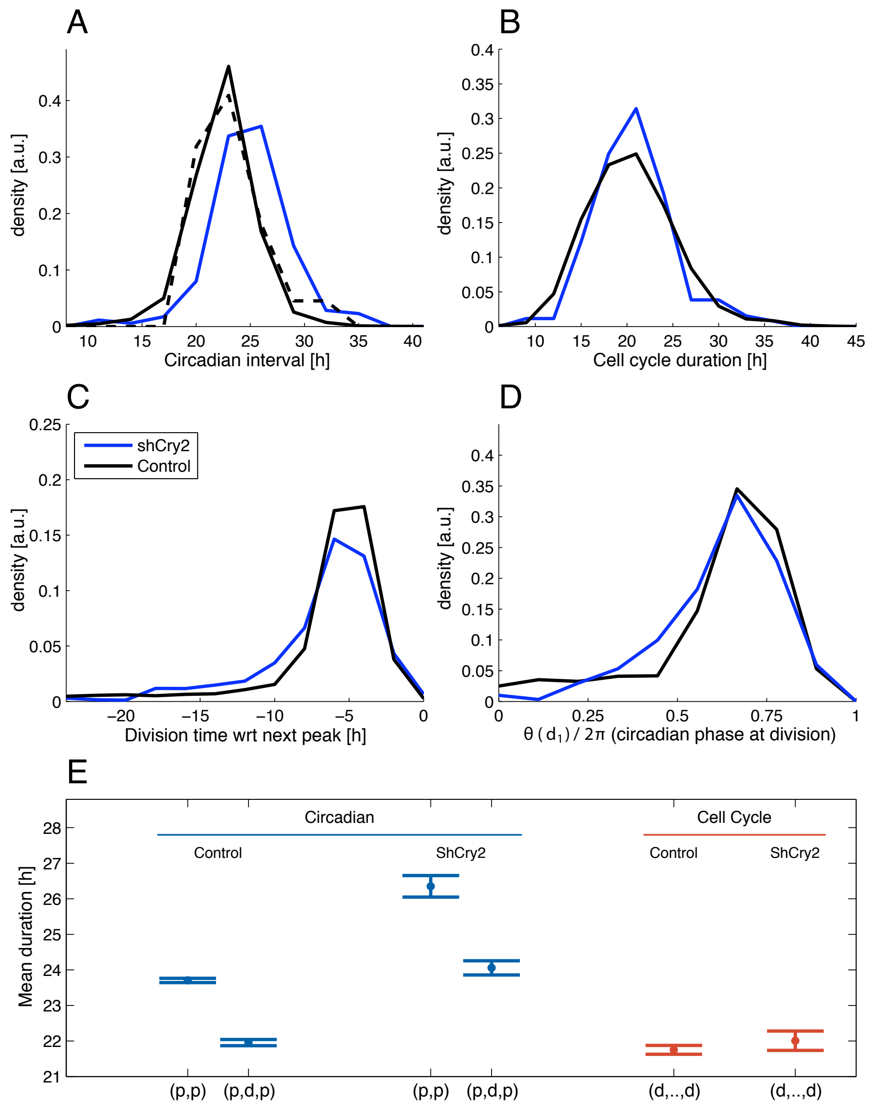

Figure S10. Cry2 deficient cells with longer circadian periods.

A. Circadian intervals (p_1,p_2) in shCry2 cells are significantly longer than controls ($p<0.02$ for Scramble shRNA cells in dashed black and $p<10^{-16}$ for the 37 ºC dataset in solid black, t-tests). Means are 26.3 +/- 4 in shCry2 and 23.7 +/- 3.1 in the controls. B. The cell cycle duration is unaffected in shCry2 cell line ($p<0.19$, t-test). C. The intervals from divisions to the next circadian peaks (d,p) are slightly lengthened in shCry2 cell line ($p<4.8\times10^{-5}$, Kolmogorov–Smirnov test, K-S). D. The circadian phases at division are slightly advanced in shCry2 cells compared to controls (while the K-S test is not significant; $p<0.008$, bootstrapped two-sample Kuiper test). E. Mean circadian intervals with divisions are significantly shorter than intervals without divisions in both control ($p<10^{-16}$, t-test) and shCry2 cells ($p<1.3\times10^{-10}$, t-test). Mean cell cycle durations is shown in red ($p<0.19$, cf. panel B). The error bars show the standard error on the mean. The total number of shCry2 cell traces analyzed is n=549.

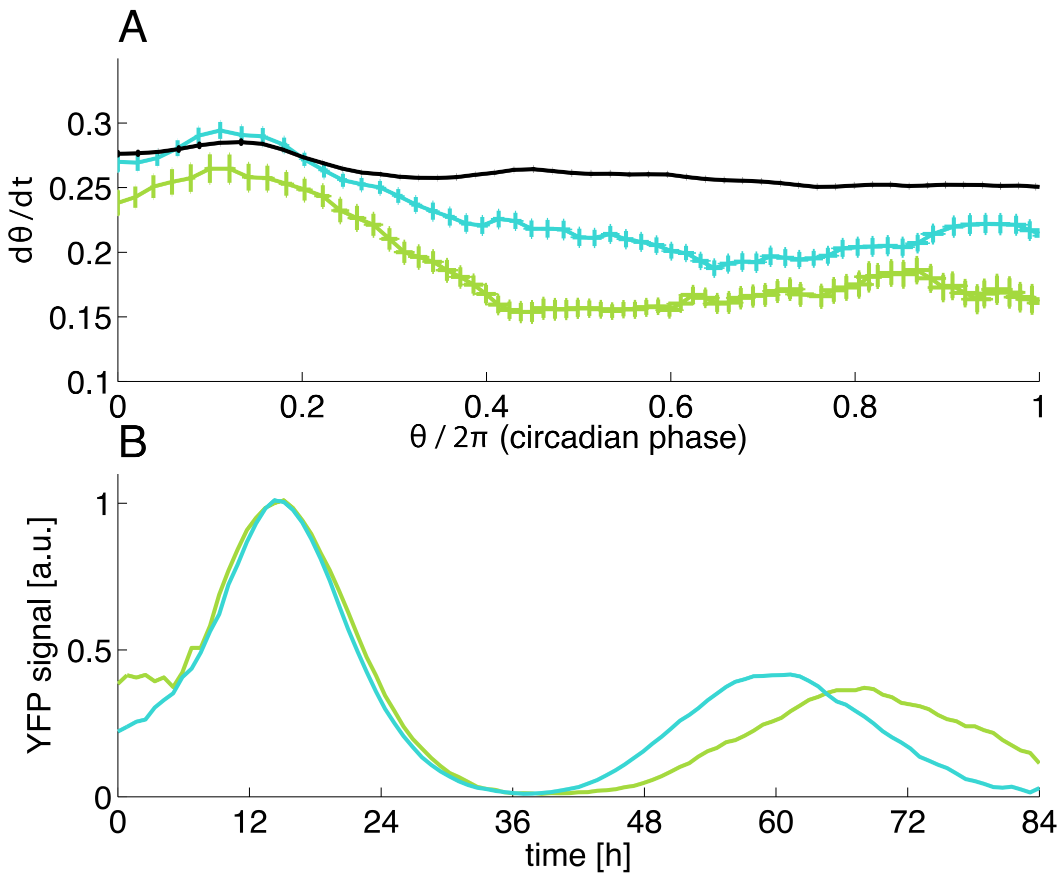

Figure S11: Instantaneous circadian phase progression under Longdaysin treatment

A. Instantaneous circadian phase velocity in intervals without divisions (p_1,p_2) is slowed down predominantly in the interval of low *Rev-Erb*α-YFP expression in dose dependent manner (Black: 37 ºC 0 μM Longdaysin, Blue: 1 μM Longdaysin, Green: 5 μM Longdaysin). B. Average of traces show that the waveform around the peak is unaffected but the 'off' interval is stretched. Standard errors on the estimated phase are shown, but are very small.

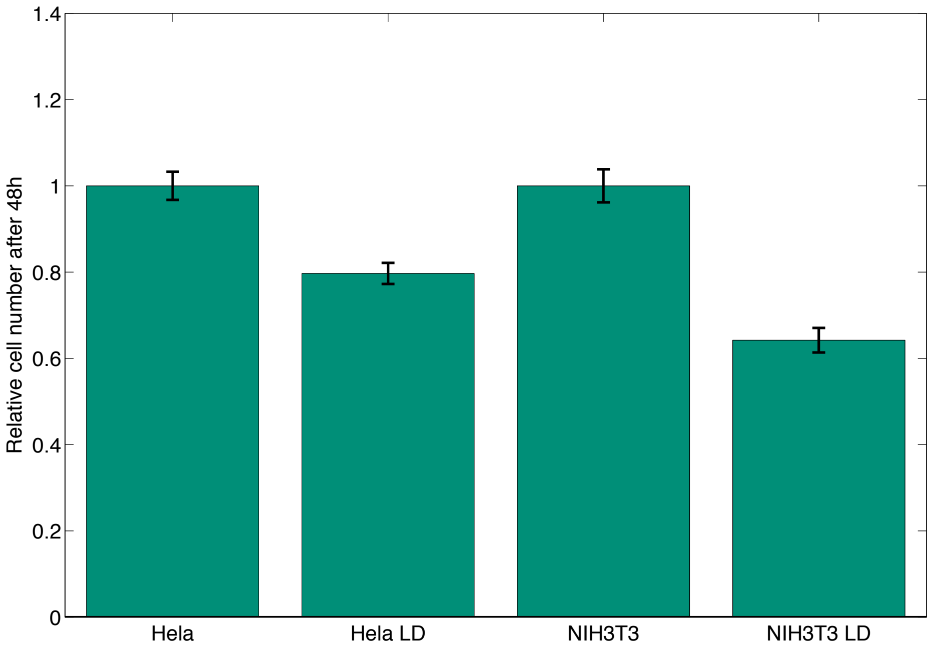

Figure S12. Longdaysin reduces cell cycle duration in NIH3T3-Venus and HeLa cells

Cell count normalized to the untreated condition after 48h of growth in presence of 5μM Longdaysin (LD) or DMSO controls. The error bars show standard error on the mean (SEM) from n=18 (HeLa DMSO), n=11 (HeLa + LD), n=18 (NIH3T3-Venus), n=18 (NIH3T3-Venus + LD) samples. The number of Longdaysin cells is significantly reduced in both cell lines ($p<1.4\ 10^{-4}$ in HeLa cells, and $p<10^{-8}$ in NIH3T3-Venus, t-tests).

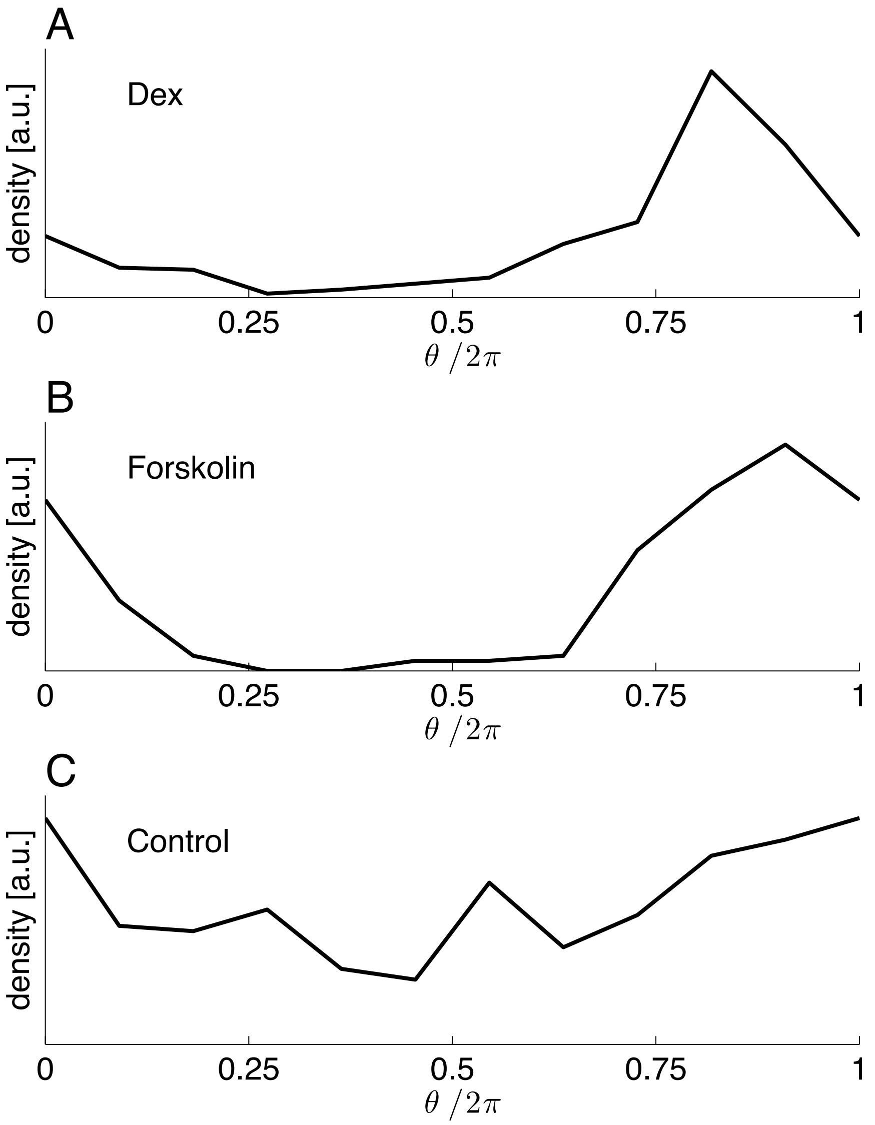

Figure S13. Initial phase distribution in dexamethasone, forskolin and control conditions.
A-C. Circadian phase distribution during the first hour after the start of the recordings. The phases were estimated as explained in Supplementary Information section IV. These distributions are clearly more peaked in the dexamethasone and forskolin treatments than in control, as expected. Synchronization indices used in Figure 7 can be computed from these distributions.
